# A Complex Tension Origin for Dilaton Gravity: Jordan Stiffness and Logarithmic Einstein Dynamics

**DOI:** 10.3390/e28050544

**Published:** 2026-05-11

**Authors:** Michaël Vaillant, Tony C. Scott

**Affiliations:** 1 Meta-Connexions, 234 Route de Seysses, 31100 Toulouse, France; michael.vaillant@meta-connexions.com; 2 Institut für Physikalische Chemie, RWTH Aachen University, 52056 Aachen, Germany

**Keywords:** scalar-tensor theory, dilaton gravity, quantum gravity, complex scalar field, Jordan frame, Einstein frame, logarithmic Schrödinger equation, Everett–Hirschman entropy

## Abstract

We propose a microphysical completion for the scalar sector of dilatonic gravity by identifying the dilaton with the coarse-grained stiffness mode of a constrained complex tension field defined on a discrete relational network. Under a controlled ordered-regime coarse-graining, the real projection of the tension scales as $\Phi \left(\Theta \right)=\Phi_{0}\;\cos\Theta$, so the Planck mass varies with the phase angle Θ and the Einstein-frame canonical scalar becomes $\varphi \propto \ln[\Phi \left(\Theta \right)/\Phi_{0}]$. This logarithmic structure emerges naturally from the Weyl map and provides the correct canonical variable for vacuum models inspired by the Logarithmic Schrödinger Equation (LogSE). We outline how this scalar–tensor interface can satisfy Solar-System constraints through environmental locking and discuss avenues for laboratory and astrophysical tests based on stiffness–coherence coupling. This paper does not introduce a new scalar–tensor EFT class as such; rather, it provides a controlled microphysical origin for a specific scalar stiffness law, Φ(Θ)∝cosΘ, and for the resulting logarithmic Einstein-frame canonical structure.

## 1. Introduction

### 1.1. Motivation: Quantizing Gravity and the Dilaton Paradigm

Formulating a consistent quantum theory of gravitation remains a central challenge in theoretical physics. Approaches that treat the entire spacetime metric as a quantum degree of freedom, such as the Wheeler–DeWitt equation arising from the Arnowitt–Deser–Misner (ADM) decomposition of general relativity, generically suffer from non-renormalizable ultraviolet divergences. As a result, many efforts have explored scalar–tensor theories, originally pioneered by Brans and Dicke [1], in which one or more scalar degrees of freedom couple to curvature. Only a subclass of scalar–tensor models is naturally described as dilaton gravity, where an auxiliary scalar (the dilaton) plays the role of an effective gravitational stiffness and is non-minimally coupled to the Ricci scalar.

In the canonical ADM reduction of specific dilaton–gravity models in 3+1 dimensions, one obtains the Logarithmic Schrödinger Equation (LogSE) for the reduced quantum dynamics [2]. In that context, the logarithmic term has been interpreted as the Everett–Hirschman entropy which bounds the sum of the entropies of a quantum state’s position and momentum probability distributions, providing a stronger bound for the Heisenberg uncertainty principle than classical variance-based principles [3,4]. It has been used as a phenomenological ingredient in superfluid vacuum theory (SVT) approaches [5,6,7].

Phenomenological routes to dilaton-like sectors also exist. For example, Modanese inserted the order-parameter field of a Bose–Einstein Condensate (BEC) into an Einstein–Hilbert-type Lagrangian (Equations (8)–(10), [8]) (see Appendix A.3.3 for more details). This illustrates a general caveat of phenomenological insertions: they can be instructive, yet the detailed structure and strength of the effective $\sqrt{-g}\;R$ coupling may depend sensitively on assumptions about the underlying microphysics.

By contrast, the 3+1 dilatonic-gravity/LogSE construction of [2] was derived via dimensional scaling and a conformal transformation, building on earlier lower-dimensional analyses [9,10,11]. The present work is complementary: we propose a microscopic origin for the stiffness/dilaton variable Φ(Θ) that appears in that continuum interface and we explain why logarithmic canonical variables arise naturally from the constrained complex tension sector.

#### Aim and Placement of the Paper

The present article addresses a limited but specific question. It is not intended as a complete discrete theory of quantum gravity, nor as a spin-network, spin-foam, or Regge discretization of spacetime geometry. Rather, it asks whether a constrained relational tension variable on a discrete substrate can generate, after coarse-graining, a scalar stiffness sector that couples to curvature in the standard scalar–tensor EFT form. Accordingly, the paper should be read neither as a full pregeometric quantum gravity theory nor as a mere reinterpretation of scalar–tensor gravity, but as a controlled microphysical completion of a scalar stiffness sector.

### 1.2. Complex Numbers as an Operationally Testable Ingredient

This subsection is included only as a structural motivation for introducing a compact phase degree of freedom on the RTQ substrate. It is not an independent derivational pillar of the gravitational construction developed below. Quantum theory is traditionally formulated over complex Hilbert spaces, yet it has long been debated whether complex numbers are physically necessary or merely a convenient representation. In particular, a “real quantum theory” obtained by restricting states and measurement operators to real numbers reproduces the predictions of a wide class of standard quantum experiments, including ordinary Bell tests.

Recent work has nevertheless shown that the complex structure becomes operationally testable in quantum networks involving independent sources. Experiments on entanglement-swapping scenarios reveal correlations that cannot be reproduced by any purely real Hilbert-space model under the standard composition postulates (tensor products for independent systems) [12]. These results concern the Hilbert-space structure of quantum theory rather than gravity per se and therefore do not directly imply any complex structure in gravitational degrees of freedom.

In the present work, this Hilbert-space discussion is used only as a structural motivation: it suggests that phase may represent a physically meaningful resource in networked quantum settings. It is not the fundamental mathematical framework of the model developed below. Our construction does not start from a Hilbert-space quantization of geometry. Instead, it starts from a constrained local state variable carried by the links of a discrete relational substrate.

At the minimal level relevant here, the only internal rotation structure explicitly used is a compact U(1) phase. Accordingly, we represent the local link state by a constrained complex tension variable, or equivalently by a two-component real field with fixed modulus. The complex notation is adopted only for economy and to make explicit the split between stiffness and coherence. The imaginary component therefore reflects a minimal phase degree of freedom in the U(1) sense used throughout this article; no larger internal symmetry structure is required for the claims made here. This economical use of a complex field should not be read as an isolated formal choice. In several cosmological constructions, a single complex scalar is used precisely as a compact representation of two coupled real degrees of freedom with distinct physical roles. For example, complex-scalar cosmologies have been used to separate amplitude-driven and phase-driven sectors, such as inflationary versus quintessence-like behavior [13,14]. In that sense, the present use of $\Lambda =\Lambda^{P}+i\Lambda^{I}$ is not introduced as an ontological excess, but as a minimal bookkeeping device for a stiffness/coherence split within a constrained U(1) structure.

### 1.3. Need for a Microphysical Completion

The absence of a microphysical derivation for the dilaton leaves open questions about the nature of the vacuum and the parameters governing its dynamics. Why does the dilaton exist, and what physical quantity does it measure? What determines its coupling to curvature, its potential, and its logarithmic behavior? Without answers to these questions, scalar–tensor theories remain phenomenological frameworks rather than derived theories. A “bottom-up” completion is therefore required, in which geometry and the dilaton emerge simultaneously from a more primitive substrate.

In particular, the persistent $H_{0}$ tension and reported (and actively debated) indications of directional dependence in late-time expansion probes—spanning X-ray cluster scaling relations [15], all-sky quasar dipole analyses [16], and low-redshift supernova tests [17,18] (the latter currently available as a preprint)—suggest that the cosmological background may exhibit spatial heterogeneity. Such directional dependence is consistent with the idea that the vacuum can carry large-scale structural anisotropies in its effective tension (or stiffness) field. Connecting these phenomenological hints to a concrete microphysical mechanism therefore motivates a more fundamental description.

While these operational theorems concern the Hilbert-space structure of quantum theory rather than gravity per se, they show that in specific network scenarios with independent sources, restricting to a real Hilbert-space model (under the standard tensor-product composition postulates) is insufficient to reproduce all quantum correlations [12,19]. We do not claim that such results derive a complex gravitational field. Instead, we use them only as a structural motivation to endow the microscopic substrate with a physically meaningful phase degree of freedom.

More generally, the literature already contains several partial precedents for the ingredients combined here. On the one hand, complex scalar fields non-minimally coupled to gravitational sectors have been studied explicitly in scalar–tensor and scalar–torsion settings [20]. On the other hand, pre-geometric graph based approaches such as Quantum Graphity have shown that continuum geometric behavior may consistently be sought as an emergent description of a fundamentally discrete relational substrate [21]. Our proposal should therefore be read neither as a standard scalar–tensor model nor as a standard graph-emergence model, but as a bridge between these two lines of thought.

The microscopic substrate introduced in this work is a network of Relational Tension Quanta (RTQ). Concretely, by “network” we mean a discrete relational structure modeled at minimal level as an oriented weighted graph, and more generally as an oriented weighted hypergraph when multi-node relations are allowed. The vertices represent RTQ nodes, while the edges (or hyperedges) encode the admissible relations and carry the local tension variables. No continuum manifold or metric is assumed at this stage.

At the minimal level used in the present article, each RTQ link carries a compact U(1) phase degree of freedom, conveniently packaged as a complex tension variable

(1) $$\Lambda =\Lambda_{0}e^{i\Theta }=\Lambda^{P}+i\Lambda^{I}.$$

Equivalently, Λ may be viewed as a two-component real field with fixed modulus and O(2) symmetry; the complex notation is adopted only for economy and to make the stiffness/coherence decomposition explicit.

In hindsight, we find ourselves appreciating the hard-earned experience of computational quantum chemistry. Though this subject deals, in principle, with fermionic formulations in the complex plane, these are transformed, decomposed or mapped unto the real plane for fast efficient (hardware) computation.

### 1.4. Our Approach: Dilaton as Stiffness of a Constrained Complex Field

In this work, we propose that the dilaton is not an independent matter field but the macroscopic representative of the intrinsic stiffness of a discrete relational network. We model the microscopic substrate as an ensemble of Relational Tension Quanta (RTQ) organized on an oriented weighted graph or hypergraph, whose connectivity encodes the fundamental relations between events. To each connection, we assign a complex tension $\Lambda =\Lambda^{P}+i\;\Lambda^{I}$, with a modulus fixed by a conservation law $\Lambda_{0}^{2}={\left(\Lambda^{P}\right)}^{2}+{\left(\Lambda^{I}\right)}^{2}$. The real part $\Lambda^{P}$ measures the network’s resistance to reconfiguration and, upon coarse-graining, gives rise to a variable effective Planck scale $\Phi \left(\Lambda \right)\propto \Lambda^{P}$. The imaginary part $\Lambda^{I}$ measures the degree of phase alignment or informational coherence. Coarse-graining over the RTQ network yields a smooth manifold description in which the dilaton emerges as the continuum stiffness field $\Phi \left(\Theta \right)=\Lambda_{0}\cos\Theta$, with the phase Θ parametrizing the distribution between physical and informational tension. A canonical scalar field $\varphi \propto \ln[\Phi \left(\Theta \right)/\Phi_{0}]$ then reproduces the logarithmic structure found in dilatonic quantum gravity, demonstrating that logarithmic dynamics arise naturally from the constraint on Λ. Our framework thus connects the dilaton paradigm to a discrete, background-free substrate and provides an explicit dictionary between the logarithmic Schrödinger equation and the angular dynamics of a complex tension field. Although the terminology is relational, the RTQ framework is not intended as a reformulation of Relational Quantum Mechanics (RQM). The relation is one of inspiration rather than identity: here, relations are introduced as microscopic substrate variables in a pre-geometric graph based model, rather than as an interpretational statement about quantum states and observers.

From this viewpoint, the present construction occupies a deliberately intermediate position. The use of a complex tension variable is formally compatible with known gravitational model-building employing complex scalar sectors [20], while the separation between a stiffness-like channel and a coherence-like channel also resonates with amplitude/phase decompositions familiar from cosmology [13,14]. At the same time, our interpretation remains more restrained: we do not identify Λ with a fundamental continuum matter field, but with a constrained local state variable living on a discrete relational substrate.

#### 1.4.1. Clarification of Scope

The scope of the present work is the scalar stiffness sector. We do not attempt to derive the full metric gravitational dynamics from RTQ combinatorics. Instead, we treat the continuum metric as an emergent IR statistical background and focus on three points: the constrained complex origin of Φ(Θ), its Jordan frame non-minimal coupling interface, and the emergence of logarithmic canonical variables in the Einstein frame. A first principles derivation of the complete gravitational sector is deferred.

In the present work, only the real projection of the constrained tension field is promoted to the Jordan frame stiffness sector. This restriction is adopted for focus and tractability, not because the imaginary projection is physically secondary. Within the fixed modulus structure $\Lambda =\Lambda^{P}+i\Lambda^{I}=\Lambda_{0}e^{i\Theta }$, the imaginary component carries the complementary coherence channel tied to the same compact phase degree of freedom. In this sense, it is not discarded; it remains structurally present through the fixed-modulus constraint, although it is not unfolded here into an explicit continuum sector. Under modulus conservation, it naturally points toward a complementary channel of coherence and relational ordering, potentially associated with informational, entropic, or topological effective dynamics. Its explicit continuum realization lies beyond the scope of the present paper.

#### 1.4.2. Interface Statement

This article is written as a micro-to-macro interface for the scalar stiffness sector. We specify a minimal compact RTQ phase-alignment toy model whose ordered-regime coarse-graining yields the infrared kinetic term for Θ(x), and we give a controlled route (under explicit ordered-graph assumptions) to an induced $\frac{1}{2}\;\Phi \left(\Theta \right)R$ coupling with Φ(Θ)∝cosΘ on the viable branch. Our technical focus is the scalar–tensor continuum interface and its Einstein-frame canonical structure; we do not attempt here a full derivation of the metric gravitational sector from RTQ microdynamics. Accordingly, no claim is made in the present article that the full Einstein dynamics of the metric have already been derived from generic RTQ combinatorics. The restricted achievement established here is the closure of the scalar stiffness channel and its controlled coupling to curvature at the EFT level. The discussion of logarithmic dynamics and the LogSE is presented as a structural bridge rather than a first-principles derivation.

#### 1.4.3. Mathematical Setting and Status of Claims

The mathematical setting used in this work is deliberately minimal. At the microscopic level, the substrate is modeled as an oriented weighted graph or hypergraph carrying a compact U(1) phase variable on each admissible relation. The local state may be written either as a constrained complex scalar $\Lambda =\Lambda_{0}e^{i\Theta }$ or, equivalently, as a two-component real field with fixed modulus. The continuum metric and scalar–tensor EFT appear only after coarse-graining in an ordered regime. Thus, the present work does not rely on a Hilbert-space construction as its fundamental framework, nor does it require any internal symmetry larger than the minimal U(1) structure stated above.

Accordingly, the claims of our work should be read with the following hierarchy. What is established here is: (i) a consistent micro-to-macro interface for the scalar stiffness mode, (ii) a controlled induced-gravity route yielding Φ(Θ)∝cosΘ at leading order on the viable branch under explicit assumptions, and (iii) the inevitability of a logarithmic Einstein-frame canonical variable once the Jordan stiffness is field-dependent. What is only motivated or proposed structurally is the bridge to LogSE-type vacuum models. What is explicitly left outside the scope of the article is a first principles derivation of the full metric gravitational sector from generic RTQ microdynamics.

##### Assumptions and Derived Statements

For clarity, we summarize the main assumptions and the status of the statements proved in this article:

**Assumption** **1** (**Ordered regime**)**.** *We assume that the RTQ network admits an ordered phase in which the phase differences $\Theta_{i}-\Theta_{j}$ on adjacent links are small and smooth on scales $L\gg \ell_{0}$. In this regime one can coarse-grain the discrete weighted Laplacian into a continuum Laplace–Beltrami operator.*

**Assumption** **2** (**Stiffness dictionary**)**.** *We adopt the ansatz $\Phi \left(\Theta \right)=\Phi_{0}\;\cos\Theta /\cos\Theta_{\mathrm{struct}}$ on the viable branch cosΘ>0 as the definition of the Jordan frame stiffness. In Appendix A we show that this functional form follows to leading order from integrating out heavy modes under Assumption 1 and suitable regularization; the normalization $\Phi_{0}=\Phi \left(\Theta_{\mathrm{struct}}\right)$ is fixed by matching to low-energy Newtonian gravity.*

**Assumption** **3** (**Kinetic and potential functions**)**.** *The Jordan frame kinetic function $K_{\Theta }\left(\Theta \right)$ and potential V(Θ) are generic positive functions; for concreteness we will study a minimal choice $K_{\Theta }=k_{0}\;\Phi$ and a confining potential $V\left(\Theta \right)=\mu^{4}{\left[\left(\cos\Theta_{\mathrm{struct}}/\cos\Theta \right)-1\right]}^{2}$. These choices ensure ghost freedom and a stable minimum near $\Theta_{\mathrm{struct}}$.*

**Assumption** **4** (**Structural bridge to the LogSE**)**.** We identify the dimensionless field $\rho \left(\Theta \right)=\Phi \left(\Theta \right)/\Phi_{0}$ with the density entering the Logarithmic Schrödinger equation and interpret the logarithmic term as arising from the angular potential associated with *Θ*. This correspondence is structural and does not constitute a derivation of the full LogSE dynamics; in particular the coefficient b and reference scale $\rho_{*}$ in Equation *(43)* remain phenomenological matching parameters.

In operational terms, the paper addresses one narrow question: how a constrained discrete tension variable can generate a scalar–tensor stiffness sector with a logarithmic Einstein-frame canonical variable. Broader extensions are discussed only as deferred prospects, not as results established here. The logic of the article is therefore the following. First, we define a minimal RTQ substrate and its constrained complex tension variable. Second, we show how an ordered-regime coarse-graining yields a scalar stiffness field and a controlled route to a Jordan-frame non-minimal coupling. Third, we study the corresponding scalar–tensor EFT and explain why the Einstein-frame canonical variable is naturally logarithmic. Phenomenological remarks are included only to indicate how this scalar sector may be constrained, not to claim a complete cosmological model.

## 2. Model Summary

For the reader’s convenience we collect here the essential ingredients of the model and summarize its fields, action, parameters and key predictions.

### 2.1. Fields

The microscopic RTQ network carries a compact phase variable $\Theta_{i}\in \left(-\pi ,\pi \right)$. Upon coarse-graining in an ordered regime, this becomes the smooth scalar field Θ(x). The Jordan frame stiffness is defined as $\Phi \left(\Theta \right)=\Phi_{0}\;\cos\Theta /\cos\Theta_{\mathrm{struct}}$. The Einstein-frame canonical scalar is defined by Equation (16). We also use the dimensionless logarithmic variable $\chi \left(\Theta \right)=\ln[\Phi \left(\Theta \right)/\Phi_{0}]$.

### 2.2. Action

The effective Jordan frame action is given in Equation (9):

$$S_{J}=\;\int \;d^{4}x\;\sqrt{-g}\left[\frac{1}{2}\;\Phi \left(\Theta \right)\;R-\frac{1}{2}\;K_{\Theta }\left(\Theta \right)\;g^{\mu \nu }\partial_{\mu }\Theta \;\partial_{\nu }\Theta -V\left(\Theta \right)\right]+S_{m}\left[g_{\mu \nu },\psi \right].$$

Kinetic and potential functions can be chosen generically; a minimal choice is $K_{\Theta }=k_{0}\;\Phi$ and $V\left(\Theta \right)=\mu^{4}{\left[(\cos\Theta_{\mathrm{struct}}/\cos\Theta )-1\right]}^{2}$.

### 2.3. Parameters

The model is specified by the reference stiffness $\Phi_{0}$ (equivalently the reduced Planck mass), the background phase $\Theta_{\mathrm{struct}}$, the kinetic parameter $k_{0}>0$ and the potential scale μ>0. Physical predictions also depend on the sign branch cosΘ>0 and the density-dependent effective potential $U_{\mathrm{eff}}\left(\Theta ;\rho_{E}\right)$.

### 2.4. Key Predictions

The universality of the coupling to matter is controlled by:

$$\alpha_{0}=\left(1/2\right)\;\tan\Theta_{\mathrm{struct}}\;{\left[\frac{3}{2}\tan^{2}\Theta_{\mathrm{struct}}+k_{0}\right]}^{-1/2}\;.$$

Solar–System bounds require $|\alpha_{0}|\;≲\;3\;\times \;10^{-3}$. Environmental locking (chameleon–like screening) occurs when $m_{\mathrm{eff}}\left(\rho \right)\;r\gg 1$, allowing the model to evade local fifth–force tests. The canonical scalar is inevitably logarithmic, making contact with LogSE–type vacuum models, but the LogSE dynamics are not derived here.

## 3. The Microscopic Substrate: RTQ Network and Constrained Tension

### 3.1. Relational Tension Quanta and Network Ontology

We posit that the fundamental constituents of spacetime are not points on a manifold but discrete relational entities, which we call Relational Tension Quanta (RTQ). At the microscopic level, the substrate is described as an oriented weighted graph G=(V,E), or more generally as an oriented weighted hypergraph H=(V,E) when relations involving more than two nodes are retained explicitly. The vertices *V* represent RTQ nodes, while the edges or hyperedges *E* encode admissible relations and support the local state variables introduced below. This type of discrete relational starting point is not without precedent in the broader emergent-spacetime literature. In particular, graph based pre-geometric models have explored how locality and continuum geometric behavior may arise only after coarse-graining from a fundamentally combinatorial substrate [21]. The present RTQ ontology differs in its use of a constrained complex tension variable on admissible relations, but it shares the same bottom-up strategy.

#### Status of the RTQ Substrate Relative to Other Discrete Approaches

In the present work, the RTQ substrate is a pregeometric relational network whose links carry local tension/alignment variables rather than continuum fields defined on a pre-existing manifold. Its role is to provide the microscopic support for an ordered coarse-grained regime in which two ingredients emerge simultaneously: an effective continuum background and a scalar stiffness channel encoded by Φ(Θ). The purpose of the paper is precisely to construct this micro-to-continuum bridge for the scalar sector.

Each RTQ represents a Planck-scale information carrier whose state updates at discrete intervals of order $t_{0}$. The relational structure is primary: links encode causal, alignment, or interaction constraints rather than distances in a pre-existing geometry. At this level there is no universal clock and no background metric. Spatial separation and temporal duration are emergent statistical descriptors reconstructed from the adjacency structure, weights, and update sequence of the RTQ network. Geometry therefore arises bottom-up from the collective dynamics of this discrete relational substrate.

### 3.2. Constrained Complex Tension and the Invariant Modulus

To each link of the RTQ network we associate a complex tension $\Lambda =\Lambda^{P}+i\;\Lambda^{I}$. This choice is also aligned with recent device independent network results showing that, under standard composition postulates, real-number quantum models cannot reproduce all quantum correlations in two-source entanglement-swapping scenarios [12,19]. We impose the fixed modulus constraint

(2) $${\left(\Lambda^{P}\right)}^{2}+{\left(\Lambda^{I}\right)}^{2}=\Lambda_{0}^{2},$$

which fixes the total “tension budget” of the network. It is convenient to parameterize the complex tension as

(3) $$\Lambda \equiv \Lambda^{P}+i\Lambda^{I}=\Lambda_{0}e^{i\Theta },\;\Lambda^{P}=\Lambda_{0}\cos\Theta ,\;\Lambda^{I}=\Lambda_{0}\sin\Theta ,$$

where the phase Θ encodes the trade-off between physical stiffness and informational coherence. Internally this modulus is dimensionless; physical units arise only when observables such as mass or charge are used to translate the dimensionless tension into SI quantities. The amplitude/phase form adopted here also has limited analogical support in the literature. In several condensed-matter and analog-gravity settings, complex order parameters provide an economical way to encode coupled degrees of freedom, with the modulus often carrying a density-like or stiffness-related interpretation [22,23]. In a related scalar-field/condensate correspondence, a complex scalar field can likewise be treated in a Gross–Pitaevskii-like form in which $\Phi^{*}\Phi$ plays a density-like role [24,25].

#### 3.2.1. Implementing the Fixed Modulus Constraint

A concrete realization starts from an unconstrained complex field Λ(x) with a steep radial potential,

(4) $$S_{\Lambda }=\int d^{4}x\sqrt{-g}\left[-\frac{Z}{2}\;g^{\mu \nu }\partial_{\mu }\Lambda^{*}\partial_{\nu }\Lambda -\frac{\kappa }{4}{\left({\left|\Lambda \right|}^{2}-\Lambda_{0}^{2}\right)}^{2}\right],\;\kappa \gg 1.$$

This is a standard EFT implementation of modulus freezing: a steep radial potential suppresses amplitude fluctuations and leaves the compact phase as the relevant low-energy degree of freedom. In the present context, the construction is used only as a controlled realization of the fixed-modulus RTQ constraint, not as an independent continuum matter model [20,24]. Writing $\Lambda =\left(\Lambda_{0}+\sigma \right)e^{i\Theta }$, the radial mode has $m_{\sigma }^{2}≃\kappa \Lambda_{0}^{2}$ and is heavy. In the EFT regime $E\ll m_{\sigma }$ one integrates out σ, obtaining $\left|\Lambda \right|≃\Lambda_{0}$ and the effective phase action $S_{\mathrm{eff}}⊃-\frac{Z\Lambda_{0}^{2}}{2}\int d^{4}x\sqrt{-g}\;{\left(\nabla \Theta \right)}^{2}+O\left(E^{2}/m_{\sigma }^{2}\right)$. Equivalently, one may impose $\left|\Lambda \right|=\Lambda_{0}$ exactly via a Lagrange multiplier $\lambda \left(x\right)\left({\left|\Lambda \right|}^{2}-\Lambda_{0}^{2}\right)$, which makes Θ the only propagating degree of freedom. The role of the fixed-modulus constraint in the consistency of the scalar sector can be summarized as follows. First, the heavy radial mode prevents uncontrolled amplitude excursions and supports the reduction to a compact phase variable at low energy. Second, because the microscopic link energy depends on 1−cos(ΔΘ), the energy stored on each RTQ link is bounded from above, so the scalar microdynamics do not develop arbitrarily large local amplitudes. Third, the identity ${\left(\Lambda^{P}\right)}^{2}+{\left(\Lambda^{I}\right)}^{2}=\Lambda_{0}^{2}$ enforces a finite tension budget: increasing the stiffness channel necessarily reduces the coherence channel, and vice versa. In this sense, the fixed-modulus mechanism is not a decorative parametrization, but the structural reason why the RTQ scalar sector remains compact, bounded, and amenable to controlled coarse-graining.

#### 3.2.2. Units and Physical Mapping

While the RTQ internal variables $\left(\Lambda_{0},\Theta \right)$ can be treated as dimensionless bookkeeping variables at the microscopic level, the continuum EFT is calibrated in SI stiffness units by the reference condition $\Phi \left(\Theta_{\mathrm{struct}}\right)=\Phi_{0}$ (cf. Equation (5)). Accordingly, once mapped to the EFT, $\Lambda_{0}$ should be understood as setting a physical stiffness/tension scale (in SI units after fixing $\Phi_{0}$), whereas Θ remains a dimensionless compact phase variable.

#### 3.2.3. Background Reference and Viability Window

In the ordered regime relevant to the IR continuum description, we treat Θ as a coarse-grained scalar field Θ(x) on the emergent manifold. We denote by $\Theta_{\mathrm{struct}}$ a homogeneous reference value (background alignment) characterizing that ordered phase and used (i) to calibrate the reference Jordan stiffness $\Phi_{0}$ (equivalently the reduced Planck mass $M_{\mathrm{Pl}}$ via Equation (8)) and (ii) to make logarithms and ratios dimensionless.

(5) $$\Phi \left(\Theta \right)=\Phi_{0}\;F\left(\Theta \right),\;F\left(\Theta \right)\equiv \frac{\cos\Theta }{\cos\Theta_{\mathrm{struct}}},$$

Herein, we work on the branch where the Jordan stiffness is positive, Φ(Θ)>0; with Φ(Θ)∝cosΘ this defines a viability window (e.g., −π/2<Θ<π/2) for the metric EFT. Approaching Θ→π/2 corresponds to Φ→0 and signals breakdown of the continuum metric description rather than a physical divergence.

We may further restrict to Θ≥0 without loss of generality, since the stiffness channel depends on cosΘ and the UV link energy depends on cos(ΔΘ), making the theory symmetric under Θ→−Θ at the level of this minimal template.

#### 3.2.4. Microphysical Origin of the Stiffness Dictionary

The stiffness dictionary (5) is used throughout the article as the IR interface between the RTQ phase field and the Jordan frame gravitational rigidity. While it can be read as a phenomenological parametrization, it can also be motivated (and, under explicit assumptions, derived) from a specified RTQ microdynamics. In an ordered RTQ regime, one may introduce a minimal set of fast internal RTQ micro-fluctuations living on the RTQ graph and integrate them out. The resulting functional determinant induces an Einstein–Hilbert term whose coefficient is controlled by a stiffness dependent UV diffusion scale of the weighted graph.

Concretely, for a weighted adjacency conductance κ(Θ) controlled by the real channel $\Lambda^{P}=\Lambda_{0}\cos\Theta$, the minimal resolvable proper time for diffusion scales as $t_{\min}\left(\Theta \right)∼\ell_{0}^{2}/\kappa \left(\Theta \right)$, hence $\Lambda_{\mathrm{UV}}^{2}\left(\Theta \right)\equiv 1/t_{\min}\left(\Theta \right)\propto \kappa \left(\Theta \right)\propto \cos\Theta$ on the viable branch. A controlled induced-gravity derivation (including an explicit error budget in the ordered phase, and the breakdown as cosΘ→0) is given in Appendix A.

#### 3.2.5. Result (Controlled)

Under the Assumptions 1 and 2 and the specific RTQ toy model described below, integrating out heavy internal modes on the weighted graph in an ordered regime induces an Einstein–Hilbert term in the continuum action with coefficient

$$\Phi \left(\Theta \right)=\Phi_{0}\;\frac{\cos\Theta }{\cos\Theta_{\mathrm{struct}}}+O\;\left(\varepsilon_{\mathrm{cg}}+\varepsilon_{R}+\varepsilon_{\Theta }\right),$$

where $\varepsilon_{\mathrm{cg}},\varepsilon_{R},\varepsilon_{\Theta }$ are small parameters controlling coarse–graining, curvature expansion and slow–variation errors as detailed in Appendix A. The functional dependence on cosΘ is robust: it follows from the spectral scaling of the weighted graph Laplacian and the proper–time cutoff, while the overall normalization is fixed by matching $\Phi \left(\Theta_{\mathrm{struct}}\right)=\Phi_{0}$.

#### 3.2.6. Scope of the Controlled Result

The derivation above should be read as a controlled result within the minimal ordered RTQ realization specified in this article. At this stage, we do not claim a general theorem covering arbitrary RTQ microdynamics. What is established is that, under Assumptions 1 and 2 and the induced-gravity route of Appendix A, the cosine stiffness law arises robustly at leading order on the viable branch. More broadly, the structure suggests that the dependence on cosΘ may reflect a wider fixed modulus ordered-phase mechanism rather than an accident of the specific toy realization used here. Establishing that stronger universality claim requires a separate treatment and lies beyond the scope of the present work.

## 4. Formulation in the Jordan Frame and Emergence of the Dilaton

### 4.1. From Discrete Adjacency to Continuous Geometry

Although the RTQ network is fundamentally discrete, a continuous spacetime description emerges when one coarse-grains over many links and update steps. At the microscopic level the dynamics are governed by a discrete action $S_{\mathrm{RTQ}}$ that sums the costs of phase misalignment between adjacent nodes together with local potentials on each node. When the orientations of links decorrelate beyond a characteristic scale $L\gg \ell_{0}$, the discrete Laplacian associated with network connectivity converges to the smooth Laplace–Beltrami operator $\nabla^{2}$ of a Riemannian or Lorentzian manifold. In this infrared limit the emergent metric $g_{\mu \nu }$ represents the statistical orientation structure of the network links, while the discrete update sequence defines a smooth time vector. The complex tension field Λ(x) is obtained by averaging the microscopic tensions over patches of many RTQ; its real part $\Lambda^{P}\left(x\right)$ and imaginary part $\Lambda^{I}\left(x\right)$ become scalar fields on the emergent manifold. In this manner, both spatial geometry and the stiffness field are “dynamically generated” from the underlying relational substrate rather than assumed a priori.

The relevance of this construction for spacetime dynamics is therefore specific but limited. In the present article, the RTQ substrate is used to motivate the existence of an emergent continuum background together with a scalar rigidity mode; it is not used to derive the full dynamical content of quantum spacetime. The dynamical output established here is the scalar stiffness channel that enters the continuum theory through Φ(Θ) and its non-minimal coupling to curvature.

### 4.2. Dilaton as Network Stiffness in the Jordan Frame

In the Jordan frame the relationship between network stiffness and spacetime geometry becomes explicit. The curvature-bearing component of the tension, $\Lambda^{P}$, is non-minimally coupled to the Ricci scalar. Using the stiffness dictionary defined in Equation (5), the effective rigidity Φ(Θ) is tied to the real projection $\Lambda^{P}$ (Equation (3)) and is positive on the EFT branch cosΘ>0.

(6) Φ(Λ)≡Φ(Θ).

The dilaton therefore arises not as an auxiliary matter field but as the continuum representative of the network’s intrinsic rigidity. Regions with larger $\Lambda^{P}$ are stiffer and correspond to a higher effective Planck mass, yielding a weaker effective gravitational coupling $G_{\mathrm{eff}}\propto 1/\Phi \left(\Lambda \right)$. Conversely, regions where $\Lambda^{P}$ is small (i.e., where coherence dominates) experience a larger susceptibility to curvature. In this picture gravity is not a fundamental force but the elastic response of the network to gradients in its stiffness field. The cosmological variation of *G* becomes a question about the phase distribution Θ(x) in the underlying network. The dilaton thus has a natural microphysical origin and its dynamics are governed by the internal kinematics of the RTQ ensemble.

### 4.3. Weyl Rescaling and the Einstein Frame

The field dependence of the stiffness can be traded from the gravitational sector to the matter sector via a conformal transformation. Performing a Weyl rescaling of the metric

(7) $$g_{\mu \nu }^{\left(E\right)}=\Omega^{2}\left(\Lambda \right)\;g_{\mu \nu }^{\left(J\right)},\;\Omega^{2}\left(\Lambda \right)=\frac{\Phi (\Lambda )}{\Phi_{0}},$$

transforms the Jordan frame action, which contains a non-minimal coupling Φ(Λ)R, into the Einstein–Hilbert form with constant reference stiffness $\Phi_{0}$ (equivalently constant reduced Planck mass $M_{\mathrm{Pl}}$). In the Einstein frame, the gravitational sector is canonical, and the field dependence now appears in a universal conformal coupling of the matter fields to the rescaled metric ${\tilde{g}}_{\mu \nu }=A^{2}\left(\Lambda \right)g_{\mu \nu }$, with $A^{2}\left(\Lambda \right)=\Omega^{-2}\left(\Lambda \right)$. While the Einstein frame is convenient for calculations, the Jordan frame remains ontologically fundamental in our framework because it directly reflects the emergence of gravity from network stiffness. The equivalence between frames underscores that the dilaton behaves as the continuum stiffness of the RTQ network, and that the variable gravitational coupling arises from the underlying phase dynamics.

## 5. Effective Field Theory and Consistency Conditions

The RTQ toy model of Section 7.5 provides a UV-regularized microscopic template for the scalar sector (compact phase and bounded link energy). In the continuum regime, gravitational phenomenology is most directly confronted in the standard covariant scalar–tensor language. Accordingly, in this work, we adopt a minimal continuum interface (EFT) in which the non-minimal stiffness coupling is written explicitly, while a complete derivation of the ΦR sector from a specified RTQ microdynamics is deferred to future work.

### 5.1. Scalar-Tensor EFT Framework and Dimensional Conventions

#### 5.1.1. Relation to Standard Scalar–Tensor Theories and Locus of Novelty

At the continuum level, the effective action introduced below belongs to the standard scalar–tensor class and is therefore not claimed as a new covariant EFT by itself. The novelty claimed in the present article lies instead in the proposed microphysical origin of the scalar stiffness variable from a constrained RTQ tension sector, in the controlled induced-gravity route to the specific law Φ(Θ)∝cosΘ on the viable branch, and in the resulting logarithmic Einstein-frame canonical structure. The manuscript should therefore be read as a microphysical completion proposal for a scalar–tensor stiffness sector, not as the introduction of a wholly new continuum action.

#### 5.1.2. Optional Translation to the Reduced Planck Mass

When comparing with particle-physics conventions, one may introduce the reduced Planck mass $M_{\mathrm{Pl}}\equiv \sqrt{\hbar c/(8\pi G_{N})}$ (SI unit: kg). It is related to the stiffness normalization by

(8) $$\Phi_{0}=\frac{c^{2}}{\hbar }\;M_{\mathrm{Pl}}^{2},\;M_{\mathrm{Pl}}^{2}=\frac{\hbar }{c^{2}}\;\Phi_{0}.$$

Therefore the present formulation is strictly equivalent to the usual $M_{\mathrm{Pl}}$-based one, but dimensionally transparent in SI. The SI dimensions and conventions used in the continuum EFT interface are summarized in Table 1.

#### 5.1.3. Dimensional Check

In SI units, the total action has the dimensions of angular momentum. Each term in Equation (9) is consistent with this unit assignment: the curvature term $\frac{1}{2}\Phi R$ has dimension $\left(\mathrm{kg}\;s^{-1}\right)\times \left(m^{-2}\right)\times \left(m^{4}\right)=J\cdot s$ when integrated over spacetime; the kinetic term $-\frac{1}{2}K_{\Theta }{\left(\nabla \Theta \right)}^{2}$ contributes $\left(\mathrm{kg}\;s^{-1}\right)\times \left(m^{-2}\right)\times \left(m^{4}\right)$; and the potential V(Θ) has dimension $J\cdot s\cdot m^{-4}$ whose integral yields J·s. The normalization chosen for Φ(Θ) and the tabulated units therefore ensures dimensional consistency of the EFT.

### 5.2. Jordan Frame EFT Template

At the continuum level, the effective action used here has the standard Jordan-frame scalar–tensor form familiar from Brans–Dicke-type and related scalar–tensor theories [1,26,27]. The specific claim of the present work is therefore not the introduction of a new continuum action class, but the proposed microphysical origin of its stiffness sector from the constrained RTQ construction. We take as a minimal Jordan frame effective action

(9) $$S_{J}=\int d^{4}x\;\sqrt{-g}\;\left[\frac{1}{2}\;\Phi \left(\Theta \right)\;R-\frac{1}{2}\;K_{\Theta }\left(\Theta \right)\;{\left(\nabla \Theta \right)}^{2}-V\left(\Theta \right)\right]+S_{m}\left[g_{\mu \nu },\psi \right],$$

where Φ(Θ) is the effective rigidity (dilaton-like stiffness mode), $K_{\Theta }\left(\Theta \right)>0$ is the Jordan frame kinetic function, and V(Θ) encodes the coarse-grained self-interaction that stabilizes the ordered phase. The stiffness dictionary adopted throughout the article is defined in Equation (5), so that $\Phi \left(\Theta_{\mathrm{struct}}\right)=\Phi_{0}$ by construction.

#### Jordan Frame Field Equations and Scalar Closure

For completeness, and to make the EFT closure explicit, we record the Euler–Lagrange equations obtained by varying the action (9) with respect to $g^{\mu \nu }$ and Θ.

(10) $$\begin{array}{cc} \Phi \left(\Theta \right)\;G_{\mu \nu }\;= & T_{\mu \nu }^{\left(m\right)}+K_{\Theta }\left(\Theta \right)\left(\partial_{\mu }\Theta \;\partial_{\nu }\Theta -\frac{1}{2}g_{\mu \nu }{\left(\nabla \Theta \right)}^{2}\right)\\ & -g_{\mu \nu }V\left(\Theta \right)+\nabla_{\mu }\nabla_{\nu }\Phi \left(\Theta \right)-g_{\mu \nu }□\Phi \left(\Theta \right). \end{array}$$

(11) $$\nabla_{\mu }\;\left(K_{\Theta }\left(\Theta \right)\nabla^{\mu }\Theta \right)+\frac{1}{2}\Phi^{\prime }\left(\Theta \right)\;R-\frac{1}{2}K_{\Theta }^{\prime }\left(\Theta \right){\left(\nabla \Theta \right)}^{2}-V^{\prime }\left(\Theta \right)=0,$$

or equivalently

(12) $$K_{\Theta }\left(\Theta \right)\;□\Theta +\frac{1}{2}K_{\Theta }^{\prime }\left(\Theta \right){\left(\nabla \Theta \right)}^{2}+\frac{1}{2}\Phi^{\prime }\left(\Theta \right)\;R-V^{\prime }\left(\Theta \right)=0.$$

Equation (12) is the generalized Klein–Gordon-like equation for the scalar stiffness field in the Jordan frame. Here

$$T_{\mu \nu }^{\left(m\right)}\equiv -\frac{2}{\sqrt{-g}}\frac{\delta S_{m}}{\delta g^{\mu \nu }},\;□\equiv g^{\mu \nu }\nabla_{\mu }\nabla_{\nu },$$

and primes denote derivatives with respect to Θ. The Einstein-frame equations follow from the Weyl map of Equation (13); they are therefore not independent of the Jordan-frame closure written above.

With Equations (10)–(12), the continuum EFT used in this article is formally closed at the Jordan-frame level. The remaining issue is therefore not one of variational consistency, but of scope: the present paper closes the scalar stiffness sector only.

### 5.3. Weyl Map to Einstein Frame and Canonical Normalization

Define the Weyl rescaling

(13) $$g_{\mu \nu }^{\left(E\right)}=\Omega^{2}\left(\Theta \right)\;g_{\mu \nu }^{\left(J\right)},\;\Omega^{2}\left(\Theta \right)=\frac{\Phi (\Theta )}{\Phi_{0}},\;A^{2}\left(\Theta \right)=\Omega^{-2}\left(\Theta \right)=\frac{\Phi_{0}}{\Phi (\Theta )}.$$

#### 5.3.1. Einstein-Frame Potential and Effective Potential

Under the Weyl map, the Jordan frame potential term transforms as

(14) $$U\left(\Theta \right)=\Omega^{-4}\left(\Theta \right)\;V\left(\Theta \right)=\frac{{\Phi_{0}}^{\;2}}{\Phi {\left(\Theta \right)}^{2}}\;V\left(\Theta \right),$$

so that U(Θ) is the Einstein-frame self-interaction. For non-relativistic matter, it is convenient to work with the conserved Einstein-frame density $\rho_{E}$, in terms of which the scalar experiences the standard screening effective potential

(15) $$U_{\mathrm{eff}}\left(\Theta ;\rho_{E}\right)=U\left(\Theta \right)+\rho_{E}\;A\left(\Theta \right),\;A\left(\Theta \right)={\left(\frac{\Phi_{0}}{\Phi (\Theta )}\right)}^{1/2}.$$

If one instead uses the Jordan frame physical density $\rho_{J}$, the two are related by the usual conformal scaling for non-relativistic matter, $\rho_{E}=A^{3}\left(\Theta \right)\;\rho_{J}$.

#### 5.3.2. Canonical Normalization

In the Einstein frame, one obtains the canonical Einstein–Hilbert term plus a scalar with a kinetic sector that receives (i) the universal Weyl contribution and (ii) the transformed Jordan kinetic term. Introducing a canonically normalized field φ, one finds the standard scalar–tensor relation

(16) $${\left(\frac{d\varphi }{d\Theta }\right)}^{2}=\Phi_{0}\left[\frac{3}{2}{\left(\frac{d\ln\Phi }{d\Theta }\right)}^{2}+\frac{K_{\Theta }\left(\Theta \right)}{\Phi (\Theta )}\right].$$

Equation (16) is the standard scalar–tensor canonical normalization obtained after Weyl rescaling from the Jordan frame to the Einstein frame [26,27,28]. In the present article, its importance is that, once combined with the RTQ stiffness law Φ(Θ)∝cosΘ, it enforces the logarithmic Einstein-frame variable discussed later. With Φ(Θ)∝cosΘ one has dlnΦ/dΘ=−tanΘ, hence

(17) $${\left(\frac{d\varphi }{d\Theta }\right)}^{2}=\Phi_{0}\left[\frac{3}{2}\tan^{2}\Theta +\frac{K_{\Theta }\left(\Theta \right)}{\Phi (\Theta )}\right].$$

#### 5.3.3. Dimensionless Coupling Convention

Since φ carries SI dimension ${\left(\mathrm{kg}\cdot s^{-1}\right)}^{1/2}$, it is convenient to introduce a dimensionless canonical variable

(18) $$\phi \equiv \frac{\varphi }{\sqrt{\Phi_{0}}}.$$

Dividing Equation (16) by $\Phi_{0}$ yields the equivalent dimensionless form

(19) $${\left(\frac{d\phi }{d\Theta }\right)}^{2}=\frac{3}{2}{\left(\frac{d\ln\Phi }{d\Theta }\right)}^{2}+\frac{K_{\Theta }\left(\Theta \right)}{\Phi (\Theta )}.$$

The corresponding dimensionless scalar coupling to matter (fifth force strength) is then defined by

(20) $$\alpha \left(\phi \right)\equiv \frac{d\ln A}{d\phi }=-\frac{1}{2}\frac{d\ln\Phi }{d\phi }=-\frac{1}{2}\;\frac{\left(d\ln\Phi /d\Theta \right)}{\left(d\phi /d\Theta \right)}.$$

Existing Solar-System and laboratory bounds therefore translate into constraints on α(ϕ) evaluated near the appropriate background value $\Theta_{\mathrm{struct}}$, and on the screening/high-density locking governed by the Einstein-frame effective potential $U_{\mathrm{eff}}\left(\Theta ;\rho_{E}\right)$ in Equation (15).

### 5.4. Consistency Conditions and Domain of Validity

The EFT (9) is viable only on a branch where the effective rigidity is positive,

(21) Φ(Θ)>0⟹cosΘ>0.

A natural viability window is therefore −π/2<Θ<π/2; one may further restrict to Θ≥0 without loss of generality in the present minimal template because Φ(Θ)∝cosΘ and the UV link energy depends on cos(ΔΘ), making the theory symmetric under Θ→−Θ.

Ghost and gradient stability require

(22) $$K_{\Theta }\left(\Theta \right)>0,\;{\left(\frac{d\varphi }{d\Theta }\right)}^{2}>0,$$

and stability of the ordered phase requires that V(Θ) admits a minimum near $\Theta_{\mathrm{struct}}$ with a positive Einstein-frame mass squared, $m_{\varphi }^{2}>0$, so that small perturbations are well behaved. The detailed form of V(Θ) (and of $K_{\Theta }$) controls screening/high-density locking and is left generic in this article; simple confining choices that grow rapidly as Θ→π/2 provide an EFT mechanism preventing the field from reaching the Φ→0 boundary within the regime of validity.

#### Strong Coupling and Breakdown

The stiffness dictionary Φ(Θ)∝cosΘ implies that Φ→0 as Θ→π/2. At the same time the microscopic UV scale $\Lambda_{\mathrm{UV}}\left(\Theta \right)$ obtained in Appendix A also scales as $\Lambda_{\mathrm{UV}}\propto \cos^{1/2}\Theta$, so the derivative expansion underlying the continuum EFT breaks down before any divergence is reached. Near Θ→π/2 the canonical normalization Equation (16) behaves as $d\varphi /d\Theta \propto \sqrt{\Phi_{0}}\;\tan\Theta$, so fluctuations of Θ become strongly coupled. The confining potential chosen in Equation (26) ensures that classical trajectories remain within the viability window and the field is driven back toward $\Theta_{\mathrm{struct}}$. Consequently the breakdown of the metric description at cosΘ→0 reflects the failure of the continuum approximation rather than a physical singularity: the underlying RTQ dynamics remain finite because the total tension budget $\Lambda_{0}^{2}={\left(\Lambda^{P}\right)}^{2}+{\left(\Lambda^{I}\right)}^{2}$ is conserved.

### 5.5. A Minimal Stabilizing Choice (Explicit and Constraint-Ready)

To make the EFT interface in Section 5 fully explicit (and directly comparable to scalar–tensor bounds), it is useful to consider a minimal stabilizing choice for the kinetic function and the Jordan frame potential.

#### 5.5.1. Minimal Kinetic Choice

We take

(23) $$K_{\Theta }\left(\Theta \right)=k_{0}\;\Phi \left(\Theta \right),\;k_{0}>0,$$

so that $K_{\Theta }\left(\Theta \right)/\Phi \left(\Theta \right)=k_{0}$ is constant. In this case, Equation (19) becomes

(24) $${\left(\frac{d\phi }{d\Theta }\right)}^{2}=\frac{3}{2}\tan^{2}\Theta +k_{0}\;.$$

The dimensionless matter coupling (20) then reduces to the closed form

(25) $$\begin{array}{ccc} \alpha (\phi )(\Theta ) & = & \frac{1}{2}\;\frac{\tan\Theta }{\sqrt{\frac{3}{2}\tan^{2}\Theta +k_{0}}},\\ \alpha_{0}\equiv \alpha \left(\phi \right)\left(\Theta_{\mathrm{struct}}\right) & = & \frac{1}{2}\;\frac{\tan\Theta_{\mathrm{struct}}}{\sqrt{\frac{3}{2}\tan^{2}\Theta_{\mathrm{struct}}+k_{0}}}\;. \end{array}$$

Hence $k_{0}$ provides a simple handle for ensuring that $\alpha_{0}$ is small enough to satisfy Solar-System fifth force constraints (“not trivially excluded”).

#### 5.5.2. A Confining Jordan Frame Potential with a Minimum at $\Theta_{\mathrm{struct}}$

We choose a minimal stabilizing potential

(26) $$V\left(\Theta \right)=\mu^{4}{\left(\frac{\cos\Theta_{\mathrm{struct}}}{\cos\Theta }-1\right)}^{2},$$

which satisfies $V(\Theta_{\mathrm{struct}})=0$, has a minimum at $\Theta =\Theta_{\mathrm{struct}}$, and diverges as cosΘ→0, thereby confining the field away from the Φ→0 boundary inside the EFT branch.

Using Equation (14), the corresponding Einstein-frame self-interaction reads

(27) $$U\left(\Theta \right)=\frac{{\Phi_{0}}^{\;2}}{\Phi {\left(\Theta \right)}^{2}}\;V\left(\Theta \right)=\mu^{4}\;\frac{\cos^{2}\Theta_{\mathrm{struct}}}{\cos^{2}\Theta }{\left(\frac{\cos\Theta_{\mathrm{struct}}}{\cos\Theta }-1\right)}^{2},$$

and the screening effective potential is $U_{\mathrm{eff}}\left(\Theta ;\rho_{E}\right)=U\left(\Theta \right)+\rho_{E}A\left(\Theta \right)$ with $A\left(\Theta \right)={\left(\Phi_{0}/\Phi \left(\Theta \right)\right)}^{1/2}$ as in Equation (15).

#### 5.5.3. Einstein-Frame Mass at the Minimum

At $\rho_{E}=0$, the minimum is at $\Theta =\Theta_{\mathrm{struct}}$ and one may estimate the Einstein-frame mass of the canonical scalar by

(28) $$m_{\varphi }^{2}={\left.\frac{d^{2}U}{d\varphi^{2}}\right|}_{\Theta_{\mathrm{struct}}}={\left.\frac{1}{{\left(\frac{d\varphi }{d\Theta }\right)}^{2}}\;\frac{d^{2}U}{d\Theta^{2}}\right|}_{\Theta_{\mathrm{struct}}}.$$

For the potential (26) one finds ${dU/d\Theta |}_{\Theta_{\mathrm{struct}}}=0$ and

(29) $$m_{\varphi }^{2}=\frac{2\mu^{4}\tan^{2}\Theta_{\mathrm{struct}}}{\Phi_{0}\left(\frac{3}{2}\tan^{2}\Theta_{\mathrm{struct}}+k_{0}\right)}.$$

This provides a minimal, fully explicit “constraint-ready” parameter set $\left(\Theta_{\mathrm{struct}},k_{0},\mu \right)$ that can be mapped to bounds on $\left(\alpha_{0},m_{\varphi }\right)$ and to environmental locking through $U_{\mathrm{eff}}\left(\Theta ;\rho_{E}\right)$.

### 5.6. Minimal Confrontation with Local Constraints

#### 5.6.1. PPN Bound (Long Range Regime)

In generic scalar–tensor theories with a universal conformal factor A(ϕ), the post-Newtonian parameter γ is related to the background coupling $\alpha_{0}\equiv \alpha \left(\phi \right)\left(\Theta_{\mathrm{struct}}\right)$ by [26,28]

(30) $$\gamma -1=-\frac{2\alpha_{0}^{2}}{1+\alpha_{0}^{2}}≃-2\alpha_{0}^{2}\;\left(\alpha_{0}^{2}\ll 1\right).$$

Solar-System light-deflection measurements therefore constrain $\alpha_{0}$ in the effectively massless regime ($m_{\varphi }r\ll 1$) [29]. Using Equation (25), a simple small angle approximation around the stiffness-dominated branch gives

(31) $$\alpha_{0}≃\frac{1}{2}\;\frac{\Theta_{\mathrm{struct}}}{\sqrt{k_{0}}}\;\left(\Theta_{\mathrm{struct}}\ll 1\right),$$

so that a bound $|\alpha_{0}|\;\leq \;\alpha_{\max}$ translates into the parametric requirement

(32) $$k_{0}≳\frac{\Theta_{\mathrm{struct}}^{2}}{4\;\alpha_{\max}^{2}}.$$

#### 5.6.2. Time Variation of *G*

The background drift obeys the identity (cf. the stiffness dictionary)

(33) $$\frac{\dot{G}}{G}{|}_{\mathrm{bg}}=\tan\left(\Theta_{\mathrm{struct}}\right)\;{\dot{\Theta }}_{\mathrm{struct}},$$

so any empirical limit on $\dot{G}/G$ bounds the admissible slow-roll of the background phase $\Theta_{\mathrm{struct}}\left(t\right)$ [30,31].

#### 5.6.3. Environmental Locking (Screening)

Recall from Equation (15) that in a non-relativistic environment described by a conserved Einstein-frame density $\rho_{E}$, the phase experiences the effective potential $U_{\mathrm{eff}}\left(\Theta ;\rho_{E}\right)=U\left(\Theta \right)+\rho_{E}A\left(\Theta \right)$ with $A\left(\Theta \right)={\left(\Phi_{0}/\Phi \left(\Theta \right)\right)}^{1/2}$. Since A(Θ) increases as Φ(Θ) decreases (i.e., as cosΘ decreases), the matter term $\rho_{E}A\left(\Theta \right)$ dynamically biases the minimum toward smaller Θ (stiffer branch), thereby reducing α(ϕ) locally. The corresponding density dependent effective mass follows from

(34) $$m_{\mathrm{eff}}^{2}\left(\rho_{E}\right)={\left.\frac{d^{2}U_{\mathrm{eff}}}{d\varphi^{2}}\right|}_{\Theta_{\min}\left(\rho_{E}\right)}={\left.\frac{1}{{\left(\frac{d\varphi }{d\Theta }\right)}^{2}}\;\frac{d^{2}U_{\mathrm{eff}}}{d\Theta^{2}}\right|}_{\Theta_{\min}\left(\rho_{E}\right)},$$

so that increasing $\rho_{E}$ generically increases the curvature of $U_{\mathrm{eff}}$ and suppresses spatial gradients of Θ (thin shell/chameleon-like behavior). This provides a minimal screening route compatible with the EFT branch cosΘ>0.

### 5.7. Numerical Viability Window (Solar-System Baseline)

#### 5.7.1. Cassini Bound on the Long Range Coupling

In the effectively massless regime ($m_{\varphi }r\ll 1$ on Solar-System scales), the PPN relation (30) [26,28] combined with the Cassini Shapiro-delay measurement $\gamma =1+\left(2.1\pm 2.3\right)\times 10^{-5}$ [29] implies (we conservatively take the quoted 1σ uncertainty as an upper limit on deviations from GR),

(35) $$\left|\gamma -1\right|≲2.3\times 10^{-5}\;⟹\;\left|\alpha_{0}\right|≲3.4\times 10^{-3},$$

where $\alpha_{0}\equiv \alpha \left(\phi \right)\left(\Theta_{\mathrm{struct}}\right)$. In the minimal kinetic choice $K_{\Theta }=k_{0}\Phi$ and for $\Theta_{\mathrm{struct}}\ll 1$, Equation (31) gives

(36) $$|\alpha_{0}|≃\frac{1}{2}\frac{\Theta_{\mathrm{struct}}}{\sqrt{k_{0}}}\;\Rightarrow \;k_{0}≳\frac{\Theta_{\mathrm{struct}}^{2}}{4\;\alpha_{\max}^{2}}\;\;\mathrm{with}\;\;\alpha_{\max}∼3.4\times 10^{-3}.$$

As a concrete illustration, $\Theta_{\mathrm{struct}}=5\times 10^{-2}$ and $k_{0}=10^{2}$ give $|\alpha_{0}|≃2.5\times 10^{-3}$, consistent with (35).

#### 5.7.2. Bound on the Background Drift of *G*

Lunar Laser Ranging analyses constrain a possible time variation of Newton’s constant at the level $\dot{G}/G_{0}=\left(-0.7\pm 3.8\right)\times 10^{-13}\;{\mathrm{yr}}^{-1}$ [32] (which is consistent with other LLR summaries [30,31]), which we translate using (33) into

(37) $$\left|{\dot{\Theta }}_{\mathrm{struct}}\right|\;≲\;\frac{4\times 10^{-13}\;{\mathrm{yr}}^{-1}}{\tan(\Theta_{\mathrm{struct}})}\;\left(\mathrm{LLR}\;\mathrm{baseline}\right).$$

This shows that the model is not only constrained by the instantaneous coupling $\alpha_{0}$, but also by the admissible slow-roll of the background phase.

#### 5.7.3. Range Requirement vs. Screening

If the scalar is unscreened, consistency on a length scale *r* requires Yukawa suppression $m_{\varphi }r≳1$; for instance r∼1AU corresponds to $m_{\varphi }≳10^{-18}\;\mathrm{eV}$. In our framework, even if the vacuum mass is smaller, the density dependent effective potential $U_{\mathrm{eff}}\left(\Theta ;\rho_{E}\right)=U\left(\Theta \right)+\rho_{E}A\left(\Theta \right)$ generically drives the minimum toward smaller Θ (stiffer branch) and increases the local curvature of $U_{\mathrm{eff}}$, hence raising $m_{\mathrm{eff}}\left(\rho_{E}\right)$ and suppressing gradients (environmental locking). Operationally, local viability is ensured whenever

(38) $$m_{\mathrm{eff}}\left(\rho_{\mathrm{local}}\right)\;r_{\mathrm{local}}\gg 1\;\mathrm{and}\;{\left|\alpha \left(\phi \right)\right|}_{\mathrm{local}}\ll 1,$$

while cosmological environments may remain in a lighter (less locked) regime.

### 5.8. Existing Bounds and Applicability

In the continuum interface of Equation (9), matter couples universally through the conformal factor $A\left(\Theta \right)={\left(\Phi_{0}/\Phi \left(\Theta \right)\right)}^{1/2}$. At the level of this minimal template the coupling is composition independent, so the most direct constraints are those on universal fifth forces, deviations from the gravitational inverse-square law, post-Newtonian parameters in the Solar System, and strong field radiative effects in compact binaries. In the unscreened regime (effectively massless on the relevant scale, $m_{\varphi }r\ll 1$), Solar-System PPN tests constrain the background coupling $\alpha_{0}$ through Equation (30) and the Cassini bound discussed in Section 5.7. For shorter ranges, torsion-balance and inverse-square-law searches constrain Yukawa-type deviations from Newtonian gravity over sub-meter scales; translating those bounds to the present scalar-tensor variables depends on the effective range $m_{\mathrm{eff}}^{-1}$ and on whether the environment is screened [33]. On much larger (orbital) scales, timing of relativistic binaries (notably binary pulsars) constrains scalar–tensor couplings through dipolar radiation and strong-field effects [28]. Cosmological probes (BBN, CMB and late-time structure) limit percent-level variations of the effective gravitational coupling between nucleosynthesis and today, implying that any cosmological drift of the background phase $\Theta_{\mathrm{struct}}\left(t\right)$ must remain slow at late times [27].

Environmental screening modifies the applicability of the above bounds. In dense regions the effective potential $U_{\mathrm{eff}}\left(\Theta ;\rho_{E}\right)$ biases the field toward smaller Θ (stiffer branch), suppresses the local coupling |α(ϕ)|, and increases the local curvature of the effective potential, raising the effective mass $m_{\mathrm{eff}}\left(\rho \right)$ (Section 5.6). Operationally, local fifth force constraints are efficiently evaded whenever the screening conditions of Equation (38) hold, $m_{\mathrm{eff}}\left(\rho_{\mathrm{local}}\right)\;r_{\mathrm{local}}\gg 1$ and ${\left|\alpha \left(\phi \right)\right|}_{\mathrm{local}}\ll 1$, while cosmological environments may remain in a lighter (less locked) regime. A systematic mapping of existing laboratory, Solar-System and binary-pulsar bounds onto the minimal parameter set $\left(\Theta_{\mathrm{struct}},k_{0},\mu \right)$ is left for future work; the purpose of the present section is to identify which constraint classes apply in the screened versus unscreened regimes and how they enter through α(ϕ) and $m_{\mathrm{eff}}\left(\rho \right)$.

## 6. Logarithmic Dynamics and the LogSE

### 6.1. Scope of the LogSE Correspondence

We do not attempt to rederive the full ADM reduction leading to the LogSE; that derivation is technically involved and is presented in detail in [2]. Our contribution is instead to provide a microphysical completion for the stiffness/dilaton sector and to clarify why the canonical Einstein-frame degree of freedom naturally involves a logarithm of Φ.

### 6.2. Canonical Reparameterization of Stiffness

The transition from the Jordan frame, where the effective Planck rigidity is field-dependent, to the Einstein frame requires a non-linear field redefinition to obtain canonical kinetic terms. In the EFT interface, the canonical field φ is defined by the scalar–tensor normalization Equation (16) (or equivalently by Equation (19) for the dimensionless field ϕ). The Weyl map generically induces an additional Einstein-frame scalar kinetic contribution involving derivatives of Φ. This motivates introducing logarithmic reparameterizations of the stiffness. The lemma below makes this statement precise and exhibits the universal ${\left(\partial \ln\Phi \right)}^{2}$ structure.

To make the link with the stiffness dictionary explicit, it is useful to introduce the logarithmic stiffness variable

(39) $$\chi \left(\Theta \right)\equiv \ln\;\left[\frac{\Phi (\Theta )}{\Phi_{0}}\right]=\ln\;\left[\frac{\cos\Theta }{\cos\Theta_{\mathrm{struct}}}\right],$$

which is dimensionless by construction. A closed-form parametrization of the canonical field can then be written as

(40) $$\varphi \left(\Theta \right)\;\approx \;\beta \;\chi \left(\Theta \right),\;\mathrm{equivalently}\;\phi \left(\Theta \right)\;\approx \;\beta_{\phi }\;\chi \left(\Theta \right),\;\;\beta_{\phi }\equiv \beta /\sqrt{\Phi_{0}}.$$

Here, β (or $\beta_{\phi }$) collects the overall canonical normalization. Importantly, this compact logarithmic form should be read as a convenient parametrization (e.g., when the Weyl-induced term dominates the canonical normalization or when $K_{\Theta }\left(\Theta \right)/\Phi \left(\Theta \right)$ varies slowly near $\Theta_{\mathrm{struct}}$), not as an independent definition: the canonical mapping is always fixed by Equation (16). Alternative canonical parametrizations may of course be introduced, for example by choosing different local field variables adapted to specific regimes or to particular forms of $K_{\Theta }\left(\Theta \right)$. What is invariant, however, is the Weyl-generated logarithmic structure encoded in ${\left(\partial \ln\Phi \right)}^{2}$. Accordingly, different parametrizations may reorganize the effective scalar dynamics, but they do not remove the basic reason why a logarithmic Einstein-frame variable is natural in the present construction. The claim made here is therefore one of structural inevitability of the logarithmic canonical sector, not uniqueness of every possible higher-level effective parametrization.

For small fluctuations around $\Theta_{\mathrm{struct}}$, one has

(41) $$\delta \chi ≃-\tan\Theta_{\mathrm{struct}}\;\delta \Theta ,\;\delta \varphi ≃-\beta \;\tan\Theta_{\mathrm{struct}}\;\delta \Theta ,\;\delta \phi ≃-\beta_{\phi }\;\tan\Theta_{\mathrm{struct}}\;\delta \Theta .$$

**Lemma** **1** (Inevitability of the logarithmic canonical field)**.** *Passing from a Jordan frame action with a non-minimal coupling $\frac{1}{2}\Phi \left(\Theta \right)\;R$ to the Einstein frame via the Weyl rescaling $g_{\mu \nu }^{\left(E\right)}=\Omega^{2}\;g_{\mu \nu }^{\left(J\right)}$ with $\Omega^{2}=\Phi /\Phi_{0}$ generically produces an Einstein-frame scalar kinetic contribution of the form*

(42) $$S⊃-\frac{3\Phi_{0}}{4}\int d^{4}x\;\sqrt{-g_{E}}\;g_{E}^{\mu \nu }\;\partial_{\mu }\;\ln\;\Phi \;\partial_{\nu }\;\ln\;\Phi \;+\;\cdots .$$

*Therefore, up to model-dependent contributions from the Jordan frame kinetic function, the canonically normalized Einstein-frame scalar necessarily involves lnΦ. With Φ(Θ)∝cosΘ, this enforces an unavoidable lncosΘ structure (equivalently $\ln[\cos\Theta /\cos\Theta_{\mathrm{struct}}]$), which is the core reason the LogSE bridge below is structurally natural rather than merely suggestive.*

**Proof.**  Starting from the Jordan frame scalar–tensor action $\int \;d^{4}x\;\sqrt{-g}\;\frac{1}{2}\Phi \left(\Theta \right)\;R$ and performing a Weyl rescaling $g_{\mu \nu }^{\left(E\right)}=\Omega^{2}\left(\Theta \right)\;g_{\mu \nu }$ with $\Omega^{2}=\Phi /\Phi_{0}$, the Ricci scalar transforms as $R=\Omega^{-2}\;\left[R_{E}-6\nabla_{E}^{2}\ln\Omega -6{\left(\nabla_{E}\ln\Omega \right)}^{2}\right]$ and the volume element transforms as $\sqrt{-g}=\Omega^{-4}\sqrt{-g_{E}}$. Substituting these relations and integrating by parts one obtains in the Einstein frame

$$\frac{1}{2}\;\int \;d^{4}x\sqrt{-g}\;\Phi \left(\Theta \right)\;R\;=\;\frac{\Phi_{0}}{2}\;\int \;d^{4}x\sqrt{-g_{E}}\;R_{E}\;-\;\frac{3\Phi_{0}}{4}\;\int \;d^{4}x\sqrt{-g_{E}}\;g_{E}^{\mu \nu }\;\partial_{\mu }\ln\Phi \;\partial_{\nu }\ln\Phi .$$

The first term yields the canonical Einstein–Hilbert action with constant stiffness $\Phi_{0}$. The second term provides an additional kinetic contribution for Θ proportional to ${\left(\partial \ln\Phi \right)}^{2}$. Combining this with the Weyl-rescaled Jordan kinetic term $-\frac{1}{2}K_{\Theta }\left(\Theta \right){\left(\nabla \Theta \right)}^{2}$ leads to the scalar–tensor normalization Equation (16), which defines the Einstein-frame canonical field. In the present model Φ(Θ)∝cosΘ, so dlnΦ/dΘ=−tanΘ and Equation (16) reduces to Equation (17), with the first term originating from the Weyl map. This demonstrates the statement of the lemma. □

### 6.3. Correspondence with Superfluid Vacuum Models (SVT)

Dilaton gravity quantized in the canonical approach leads to an effective LogSE [2]. The logarithmic non-linearity has been discussed as a route to a small but non-zero vacuum energy scale in LogSE-based vacuum models [34], and MOND-like phenomenology at galactic scales has been analyzed in SVT/LogSE contexts [6,7]. Furthermore, the log term in LogSE is absolutely essential for superfluids according to precise experimental data at very low temperatures, around 1K [35], and since it is not Lipschitz continuous when its argument is zero, may even contribute to the spontaneous symmetry breaking phenomenon of the Higgs boson [34,36]. As we can see, the presence of the LogSE for both cosmological and quantum Bose systems reflects Volovik’s notion that microscopic phenomena are reflected in the macroscopic universe [23].

In our framework, this non-linearity has a natural structural origin for the logarithmic degree of freedom. More broadly, this interpretation is conceptually aligned with a family of approaches in which a complex order parameter separates an amplitude-related structural sector from a phase-related coherence sector [22,23]. In such analogical settings, amplitude variables govern density or rigidity, while phase variables govern collective order, transport, and topological organization. The present RTQ construction does not identify the vacuum with any specific condensed-matter medium, but it exploits an analogous structural split.

#### Scope Note: Structural Correspondence Rather than a First Principles LogSE Derivation

The appearance of a logarithmic canonical variable is a robust consequence of the Weyl map (Lemma 1) and the stiffness dictionary Φ(Θ)∝cosΘ. In contrast, identifying the logarithmic variable with a specific non-linear Schrödinger dynamics is presented here as an EFT-level bridge. Concretely, LogSE models may be formulated from an effective energy functional of the schematic form

(43) $$E\left[\psi \right]=\int d^{3}x\left[\frac{\hbar^{2}}{2m}{\left|\nabla \psi \right|}^{2}+b{\left|\psi \right|}^{2}\ln\;\left(\frac{{\left|\psi \right|}^{2}}{\rho_{*}}\right)+\cdots \right],$$

whose variation yields a logarithmic term in the corresponding Schrödinger-type equation of motion. In our framework, the natural dimensionless stiffness density is $\rho \left(\Theta \right)\equiv \Phi \left(\Theta \right)/\Phi_{0}$ and the logarithmic variable χ=lnρ arises canonically from the scalar–tensor structure. Deriving the precise LogSE coefficient *b* (and the reference scale $\rho_{*}$) from RTQ microdynamics would require computing the coarse-grained free-energy contribution associated with the informational sector ($\Lambda^{I}$) and is left for future work. The claim made here is therefore that the RTQ stiffness mode provides the correct logarithmic degree of freedom required by such vacuum models, with a microphysical derivation of the full LogSE Hamiltonian deferred.

### 6.4. An Entropic Route to the LogSE Non-Linearity (Prototype)

Let $\rho \left(x\right)\equiv {\left|\psi \left(x\right)\right|}^{2}$ denote the coarse-grained stiffness density, identified in our dictionary as $\rho =\Phi \left(\Theta \right)/\Phi_{0}$ (so $\rho_{*}\equiv \rho \left(\Theta_{\mathrm{struct}}\right)=1$). Under the fixed modulus constraint ${\left(\Lambda^{P}\right)}^{2}+{\left(\Lambda^{I}\right)}^{2}=\Lambda_{0}^{2}$, increasing local stiffness reduces the available microstate volume of the coherence sector. A minimal local encoding of this loss is the additive Shannon/Everett–Hirschman entropy functional

(44) $$S_{I}\left[\psi \right]=-k_{B}\int d^{3}x\;\rho \left(x\right)\;\ln\;\left(\frac{\rho (x)}{\rho_{*}}\right),$$

where $k_{B}T_{\mathrm{eff}}$ is an effective information-sector susceptibility (not necessarily a physical temperature). Assuming the coherence sector acts as an effective bath, the coarse-grained free-energy reads

(45) $$\begin{array}{cc} E[\psi ] & =\int d^{3}x\;\frac{\hbar^{2}}{2m}{\left|\nabla \psi \right|}^{2}-T_{\mathrm{eff}}\;S_{I}\left[\psi \right]\\ \; & =\int d^{3}x\left[\frac{\hbar^{2}}{2m}{\left|\nabla \psi \right|}^{2}+b\;{\left|\psi \right|}^{2}\ln\;\left(\frac{{\left|\psi \right|}^{2}}{\rho_{*}}\right)\right],\;b\equiv k_{B}T_{\mathrm{eff}}. \end{array}$$

Using $i\hbar \partial_{t}\psi =\delta E/\delta \psi^{*}$, one obtains

(46) $$i\hbar \;\partial_{t}\psi =\left[-\frac{\hbar^{2}}{2m}\nabla^{2}+b\;\ln\;\left(\frac{{\left|\psi \right|}^{2}}{\rho_{*}}\right)\right]\psi ,$$

where an additive constant from the variation can be absorbed into a global phase. In this EFT-level bridge, *b* and $\rho_{*}$ are matching parameters encoding coarse-grained information-sector physics.

### 6.5. Everett–Hirschman Entropy and Phase Misalignment

In the LogSE literature, the logarithmic non-linearity has been interpreted as an Everett–Hirschman entropy associated with uncertainty in phase space. Within the RTQ framework, this entropy measures the misalignment of phases among neighboring tension quanta. The entropy quantifies the geometric spreading of phase misalignment along the emergent causal/alignment structure, and—through the fixed modulus constraint $\Lambda_{0}^{2}={\left(\Lambda^{P}\right)}^{2}+{\left(\Lambda^{I}\right)}^{2}$—it ties coherence loss (decrease in $\Lambda^{I}$) to a compensating increase in stiffness $\Lambda^{P}$. In this sense, gravity is the spatial signature of coherence degradation, while entropy increase is its temporal signature. The logarithmic potential in the effective action can therefore be interpreted as the work required to convert coherence into curvature while maintaining the global tension budget. This recasts the Everett–Hirschman entropy not as an abstract measure but as the micro-geometric cost of phase misalignment in a relational network.

## 7. Quantization and the Tension Budget

### 7.1. Localizing the Quantum

Traditional approaches to quantum gravity attempt to quantize the entire metric and encounter non-renormalizable divergences. In contrast, our approach confines the quantum degrees of freedom to the complex tension field Λ and the discrete update dynamics of the RTQ network. The metric $g_{\mu \nu }$ is an emergent, coarse-grained statistical object built from network statistics and is treated classically or semiclassically. Quantization is localized within the stiffness–coherence field itself: the phase Θ and its canonical conjugate φ are the true quantum variables. In this construction, the primary quantum degrees of freedom are placed in the compact stiffness–coherence sector (rather than in the continuum metric). This avoids the specific UV pathology associated with direct quantization of the full metric in Wheeler–DeWitt-type approaches. The compactness of Θ and the bounded link energy of the RTQ toy model suggest improved UV behavior for the scalar sector; a full renormalization and unitarity analysis of the complete continuum EFT is beyond the scope of the present work.

### 7.2. The Tension Budget as a Regularization Mechanism

In classical general relativity singularities such as black holes or the Big Bang arise when curvature invariants diverge. In our framework the invariant modulus $\Lambda_{0}$ acts as a built-in regulator. As physical stiffness $\Lambda^{P}$ increases toward its maximum $\Lambda_{0}$, the coherence $\Lambda^{I}$ diminishes, indicating that the network approaches a frozen informational state. Extreme regimes are thus reached through a finite set of admissible phase states rather than continuous divergences. When $\Lambda^{P}\to \Lambda_{0}$, the system enters an informationally frozen domain where the metric description remains finite but degenerate. Further energy input cannot increase $\Lambda^{P}$ but must rotate the phase into coherence or trigger a topological transition. Classical singularities are therefore interpreted, within the present effective description, as tension-saturated (or coherence-depleted) regimes where the continuum metric EFT may cease to apply before any divergence is reached.

### 7.3. Emergence of Planck’s Constant (Structural Mapping and Observational Consistency)

#### 7.3.1. Scope of the Planck-Constant Mapping

The relation below is proposed as a structural mapping between discrete RTQ capacity and the continuum action quantum, not as a prescription for a locally varying *ℏ* in laboratory effective field theory. In the late-time EFT regime used in this work, *ℏ* is treated as a constant fixed by the background calibration.

#### 7.3.2. SI Calibration and Dimensional Bookkeeping

At the microscopic RTQ level, $\left(\Lambda_{0},\ell_{0},t_{0},\Theta \right)$ are taken as unit-free network variables. Physical units enter only after matching the IR scalar–tensor EFT to the observed gravitational calibration, which we encode by fixing the Jordan stiffness at the structural background phase, $\Phi \left(\Theta_{\mathrm{struct}}\right)=\Phi_{0}$. After this matching, $\ell_{0}$ and $t_{0}$ inherit the dimensions of length and time on the emergent metric sheet, while $\Lambda_{0}$ sets an effective stiffness/tension scale (force dimension in SI units). Consequently, the product $\Lambda_{0}\;\ell_{0}\;t_{0}$ carries the dimensions of an action.

#### 7.3.3. Normalization Choice (Not a Dynamical Law)

We introduce an emergent action scale by evaluating the coherence share of the fixed modulus budget at the structural background phase,

(47) $$\hbar \;\equiv \;\hbar \left(\Theta_{\mathrm{struct}}\right)\;:=\;\kappa \;\Lambda_{0}\;\ell_{0}\;t_{0}\;\sin\Theta_{\mathrm{struct}},$$

where κ is a dimensionless O(1) matching constant that depends on the precise microscopic convention used to translate network variables into continuum observables. Equation (47) is therefore to be read as a calibration identity fixing the constant *ℏ* of the IR EFT once the background environment is specified, rather than as a prediction for local variations of *ℏ*.

#### 7.3.4. If One Allows a Hypothetical Slow Background Drift

If, as a purely hypothetical extension, the homogeneous background phase were to drift slowly in cosmological time, a fractional change would scale as

(48) $$\frac{\dot{\hbar }}{\hbar }=\cot\left(\Theta_{\mathrm{struct}}\right)\;{\dot{\Theta }}_{\mathrm{struct}}.$$

Any effective drift in *ℏ* would in general correlate with drifts of dimensionless couplings (e.g., α) through the same calibration map, and is therefore expected to be extremely tightly bounded by precision spectroscopy and optical-clock comparisons. In the present work, we do not model such correlated drifts; we simply note that consistency with laboratory bounds motivates treating $\Theta_{\mathrm{struct}}$ as effectively constant on laboratory timescales, in line with the environmental locking assumption discussed in Section 5.6.

Within the conservative EFT reading adopted here, Equation (47) fixes the global action scale at the formation of the ordered causal sheet and does not imply a locally varying *ℏ* in the phenomenology studied in this work.

### 7.4. Dimensional Consistency and the Modulus $\Lambda_{0}$

The modulus $\Lambda_{0}$ is not a free parameter that can be tuned at will. Dimensional analysis of the effective coupling constants shows that $\Lambda_{0}$ acts as a scale-invariant pivot from which the observed constants of nature emerge. When the network dynamics are projected onto a three-dimensional manifold with update frequency $1/t_{0}$, the values of *G*, *c*, and *ℏ* are determined by specific ratios of the network cardinality and $\Lambda_{0}$. For example, the intrinsic grain $\ell_{0}$ expressed in SI units is of order $\alpha_{G}^{-1/2}\;\ell_{P}$, where $\alpha_{G}$ is a dimensionless alignment coefficient and $\ell_{P}$ is the Planck length. Thus, the Planck scale arises naturally from the finite tension budget of the network. The correlated emergence of *G*, *c*, *ℏ* and other constants implies that once $\Lambda_{0}$ and the network configuration are specified there is little freedom left; the UV completion of the theory is controlled by the internal geometry of the relational substrate rather than by external renormalization procedures.

### 7.5. A Minimal RTQ UV Toy Model (Phase Alignment) and the Continuum Interface

A key claim of this work is that the complex tension field $\Lambda =\Lambda^{P}+i\Lambda^{I}=\Lambda_{0}e^{i\Theta }$ can be treated as a microscopic network variable whose coarse-grained stiffness mode becomes dilaton-like in the IR. To make this statement constructive, we introduce a minimal UV toy model on an RTQ adjacency graph.

#### 7.5.1. Discrete Degrees of Freedom and Bounded Link Energy

Assign to each RTQ node *i* a compact phase $\Theta_{i}$ and a complex tension state $\Lambda_{i}=\Lambda_{0}e^{i\Theta_{i}}$ (with fixed modulus $|\Lambda_{i}|=\Lambda_{0}$). A natural measure of mismatch between adjacent nodes 〈i,j〉 is the squared distance in the complex plane:

(49) $${\left|\Lambda_{i}-\Lambda_{j}\right|}^{2}={\left|\Lambda_{0}e^{i\Theta_{i}}-\Lambda_{0}e^{i\Theta_{j}}\right|}^{2}=2\Lambda_{0}^{2}\left(1-\cos(\Theta_{i}-\Theta_{j})\right).$$

This immediately motivates an XY-like phase alignment energy on each link. Because Θ is compact, 1−cos(ΔΘ)∈[0,2], so the energy per link is strictly bounded. At fixed microscopic grain $\ell_{0}$ the model is UV-regularized by construction.

#### 7.5.2. Minimal Discrete Action

We consider the template action

(50) $$S_{\mathrm{RTQ}}=\sum_{\langle i,j\rangle }J_{ij}\;\left(1-\cos(\Theta_{i}-\Theta_{j})\right)+\sum_{i}U\left(\Theta_{i}\right)+S_{T}\left[T^{\mu }\right],$$

where $J_{ij}$ encodes the link stiffness capacity and U(Θ) is the microscopic alignment potential whose coarse-grained counterpart is captured by V(Θ) in the EFT interface. The term $S_{T}\left[T^{\mu }\right]$ represents the causal/alignment sector used to reconstruct an operational time direction in the ordered regime.

#### 7.5.3. Coarse-Graining and IR Kinetic Term

On length scales $L\gg \ell_{0}$, assume an ordered statistically isotropic phase in which phase variations are smooth, ΔΘ≪1 over one link. Then

(51) $$1-\cos\left(\Delta \Theta \right)≃\frac{1}{2}{\left(\Delta \Theta \right)}^{2},\;\Theta_{i}-\Theta_{j}≃\ell_{0}\;n^{\mu }\partial_{\mu }\Theta \left(x\right),$$

so that the link sum coarse-grains to the standard covariant gradient term

(52) $$\sum_{\langle i,j\rangle }J_{ij}\;\left(1-\cos(\Theta_{i}-\Theta_{j})\right)\;⟶\;\int d^{4}x\;\sqrt{-g}\;\frac{K_{\Theta }\left(\Theta \right)}{2}\;g^{\mu \nu }\partial_{\mu }\Theta \;\partial_{\nu }\Theta +\cdots ,$$

where $K_{\Theta }\left(\Theta \right)$ absorbs the coarse-grained link density, typical $J_{ij}$, and the factor $\ell_{0}^{2}$. This provides a concrete UV-to-IR route for the scalar sector without quantizing the background metric.

#### 7.5.4. Continuum Stiffness Dictionary (Dilaton-like Mode)

The real projection of the tension is $\Lambda^{P}=\Lambda_{0}\cos\Theta$. In the continuum interface used throughout the article, we encode the long wavelength stiffness response geometrically by introducing a Jordan frame effective rigidity Φ(Θ) proportional to $\Lambda^{P}$. Using the stiffness dictionary defined in Equation (5) and the microscopic projection in Equation (3), Φ(Θ) plays the role of a dilaton-like rigidity field controlling the effective gravitational response. A full derivation of the gravitational sector from a specified RTQ microdynamics is deferred to follow-up work; the purpose of this toy model is to make explicit (i) the compact UV degrees of freedom, and (ii) the emergence of a universal IR kinetic operator for Θ(x) compatible with the scalar–tensor interface.

## 8. Phenomenological Consequences and Tests

### 8.1. Effective Gravitational Coupling as Stiffness Susceptibility

In the present framework, the quantity that plays the role of a gravitational coupling is controlled by the coarse-grained stiffness field $\Phi \left(\Theta \right)\propto \Lambda^{P}=\Lambda_{0}\cos\Theta$. In the Jordan frame, this implies a local effective susceptibility

(53) $$G_{\mathrm{eff}}\left(\Theta \right)\;\propto \;\frac{1}{\Phi (\Theta )}\;\propto \;\frac{1}{\cos\Theta }.$$

For practical comparisons, to observation it is useful to normalize this relation to a reference background phase $\Theta_{\mathrm{struct}}$ (the phase characterizing the current cosmological environment),

(54) $$\frac{G_{\mathrm{eff}}\left(\Theta \right)}{G_{\mathrm{eff}}\left(\Theta_{\mathrm{struct}}\right)}\;=\;\frac{\cos\Theta_{\mathrm{struct}}}{\cos\Theta }.$$

This expression should not be read as implying that *G* “diverges” in any physical situation. Rather, as Θ→π/2, the stiffness $\Lambda^{P}\to 0$ and the smooth-manifold description is expected to break down: the effective field theory ceases to be valid before any divergence is reached. A key observational consequence is therefore not a large variation of *G*, but the existence of screening or environmental dependence in Θ(x) that keeps local deviations within current bounds while allowing non-trivial cosmological behavior.

### 8.2. Granularity: Qualitative Signatures Beyond a Sharp Planck Cutoff

A distinctive feature of the constrained complex tension picture is that the Planck domain is not introduced as an ad hoc ultraviolet cutoff. Instead, it is interpreted as the natural coarse-graining limit of the RTQ substrate. This motivates phenomenological searches for granularity signatures that can arise even if the fundamental microdynamics remain inaccessible.

Two broad classes of effects are especially natural in a network completion: (i) a scale-dependent effective dimension (spectral dimension flow) as the coarse-graining window is varied, and (ii) non-Gaussian “metric textures” at very short scales reflecting discrete adjacency statistics. Operationally, such signatures could be sought through future high-precision interferometry, cross-spectral density methods for stochastic gravitational backgrounds, or consistency tests of effective field descriptions across multiple frequency bands.

We emphasize that the present article does not attempt a complete derivation of such signatures from a specified RTQ microdynamics. Rather, it identifies the observational channels that are most directly linked to the core mechanism developed in Section 4, Section 5, Section 6 and Section 7: a stiffness field Φ(Θ) with a constrained phase degree of freedom.

### 8.3. Laboratory Probes of Stiffness–Coherence Coupling

The decomposition $\Lambda =\Lambda^{P}+i\Lambda^{I}$ suggests a pragmatic experimental question: can one bound (or detect) any coupling between macroscopic coherence resources and the effective stiffness channel Φ? A conservative way to state this is to introduce a dimensionless coupling parameter η that controls how strongly externally prepared coherence states might bias the local phase Θ away from its environmental value. This perspective aligns with proposals in relativistic quantum metrology where macroscopic coherence in BECs is used as a probe of gravitational effects (e.g., phononic excitations induced by spacetime perturbations) [37,38].

One convenient phenomenological ansatz is to expand the phase field around a background reference value $\Theta_{\mathrm{struct}}$ appropriate to the environment under consideration (laboratory/Solar-System), and to parametrize a small coherence-induced bias as

(55) $$\Theta \;=\;\Theta_{\mathrm{struct}}+\eta \;K,$$

where *K* is a dimensionless coherence proxy (to be operationally defined for a given platform) and |ηK|≪1. To leading order, this implies a fractional stiffness modulation

(56) $$\frac{\delta \Phi }{\Phi }\;≃\;-\tan\left(\Theta_{\mathrm{struct}}\right)\;\eta K,\;\frac{\delta G_{\mathrm{eff}}}{G_{\mathrm{eff}}}\;≃\;+\tan\left(\Theta_{\mathrm{struct}}\right)\;\eta K.$$

This formulation makes a clear prediction structure: experiments constrain η by bounding δΦ/Φ or $\delta G_{\mathrm{eff}}/G_{\mathrm{eff}}$ under controlled changes in *K*.

Three conservative laboratory channels can be framed as null tests:

- Precision gravimetry: search for reproducible, configuration-dependent shifts in weight correlated with controlled changes in *K* in a nearby sample.
- Clock and resonator probes: track fractional frequency shifts of high-Q resonators or clocks placed in controlled proximity to coherent media, interpreted as constraints on local conformal rescaling proxies.
- Transient protocols: search for time-dependent signatures during rapid modulation of *K*, which would correspond to $\dot{\Theta }\neq 0$ and therefore to a dynamical stiffness response.

The value of this approach is that it does not assume any large effect size. It translates the conceptual content of our framework into measurable bounds on η, and it cleanly separates speculative applications from experimentally controlled constraints.

### 8.4. Observable Consequences—Summary for Constraints and Experiments

Let $\Theta \left(x\right)=\Theta_{\mathrm{struct}}\left(t\right)+\delta \Theta \left(x\right)$ denote small departures around the ordered-phase reference.

- Solar-System baseline (unscreened regime): PPN tests constrain the background coupling $\alpha_{0}$ through Equation (30) and the Cassini bound (Equation (35)), which maps to a simple parametric requirement on $\left(\Theta_{\mathrm{struct}},k_{0}\right)$ via Equation (31) (or Equation (36)).
- Time variation of *G*: LLR limits constrain the admissible drift of the background phase via Equations (33) and (37). This bounds slow-roll evolution of $\Theta_{\mathrm{struct}}\left(t\right)$.
- Environmental locking (screening): in dense regions, the matter term in $U_{\mathrm{eff}}$ (Equation (15)) biases the minimum toward smaller Θ (stiffer branch) and increases the local curvature, leading to $m_{\mathrm{eff}}\left(\rho_{\mathrm{local}}\right)\;r_{\mathrm{local}}\gg 1$ and ${\left|\alpha \left(\phi \right)\right|}_{\mathrm{local}}\ll 1$ as summarized in Equation (38) (see also Equation (34)).
- Laboratory null tests: controlled coherence modulation constrains a possible bias δΘ≃ηK and therefore bounds any correlated fractional shifts of the effective rigidity or coupling discussed in Section 8.

## 9. Conclusions

### 9.1. Summary of the Mechanism

We have proposed a minimal microphysical completion for dilatonic gravity in which the dilaton is identified with the coarse-grained stiffness mode of a constrained complex tension field $\Lambda =\Lambda^{P}+i\Lambda^{I}$ of fixed modulus $\Lambda_{0}$. In this sense, the completion achieved here targets the scalar (dilaton-like) stiffness channel, while the explicit emergence of the full metric gravitational sector from RTQ combinatorics remains an open step beyond the present scope.

The central technical point is that the Jordan frame stiffness dictionary (Equation (5)), together with the Einstein-frame canonical normalization (Equation (16)), naturally leads to a logarithmic stiffness variable $\chi =\ln[\Phi \left(\Theta \right)/\Phi_{0}]$ (Equation (39)). In this construction, logarithmic structures familiar from the Logarithmic Schrödinger Equation (LogSE) are not inserted by hand but arise as the canonical parametrization of a constrained complex degree of freedom.

Conceptually, the framework localizes quantization in the stiffness–coherence sector rather than in the emergent background metric, and it replaces uncontrolled divergences by an invariant “tension budget” $\Lambda_{0}^{2}={\left(\Lambda^{P}\right)}^{2}+{\left(\Lambda^{I}\right)}^{2}$ that limits extreme regimes within the effective description.

### 9.2. What Is Established Here, and What Is Deferred

What is established in the present work is a minimal and internally consistent interface between a constrained RTQ tension sector and a scalar–tensor continuum description. At the continuum level, the action belongs to the standard scalar–tensor family; the point of the article is therefore not to claim a novel covariant EFT in isolation, but to provide a specific microphysical origin for the stiffness law Φ(Θ)∝cosΘ and a controlled route to its logarithmic Einstein-frame variable. More precisely, this article provides: (i) a graph based microscopic setting with a compact U(1) phase degree of freedom, (ii) a controlled leading-order route to the Jordan frame stiffness law Φ(Θ)∝cosΘ on the viable branch, and (iii) a clear reason why the Einstein-frame canonical variable is naturally logarithmic once the stiffness is field-dependent.

What is not established here is equally important. We do not derive the full metric gravitational sector from generic RTQ combinatorics; we do not claim a first-principles derivation of the full LogSE dynamics; and we do not claim universality of the present results beyond the ordered-regime assumptions and the minimal microphysical template made explicit in this article. These broader questions are deferred to follow-up work.

The intended logical placement of the present result within a broader program may be summarized schematically as

$$\begin{array}{cc} \mathrm{constrained}\;\mathrm{RTQ}\;\mathrm{tension}\;\sec\mathrm{tor} & ⟹\Phi (\Theta )\propto \cos\Theta \\ & ⟹\mathrm{scalar}–\mathrm{tensor}\;\mathrm{stiffness}\;\mathrm{EFT}\\ & ⟹\chi =\ln[\Phi \left(\Theta \right)/\Phi_{0}]\\ & ⟹\mathrm{possible}\;\mathrm{future}\;\mathrm{bosonic}/\mathrm{fermionic}/\mathrm{gauge}\;\mathrm{extensions}. \end{array}$$

In this schematic chain, only the scalar stiffness sector up to the logarithmic Einstein-frame variable is established here; the later sectors remain prospective.

Accordingly, the manuscript should be read as a scalar-sector completion paper first, with broader implications stated only as deferred prospects rather than as established results.

More broadly, the scalar stiffness mechanism developed here may be viewed as one entry point into a wider pregeometric program in which additional relativistic and quantum sectors could emerge from constrained RTQ dynamics after coarse-graining. In that broader perspective, the present logarithmic Einstein-frame structure may represent only the scalar projection of a larger architecture, whose possible extensions to Klein–Gordon, Dirac, or gauge sectors remain to be established explicitly. We do not claim such derivations here. Rather, the present article identifies a controlled sector that may serve as a foundation for a more comprehensive follow-up theory.

By comparison with nearby lines of work, standard Brans–Dicke and scalar–tensor theories formulate the scalar sector directly at the continuum level [1,26,27]; complex-scalar gravitational models explore continuum amplitude/phase structures without the present RTQ microphysical substrate [20]; dilatonic gravity connected to logarithmic quantum dynamics was developed at the continuum level in [2]; and graph-based pregeometric approaches study the emergence of continuum geometry from combinatorial structures [21]. The specific contribution of the present paper is to connect a constrained relational microvariable to a scalar stiffness law Φ(Θ)∝cosΘ and, through the standard Weyl map, to a logarithmic Einstein-frame canonical variable.

Within that scope, the contribution of the article is to turn a previously phenomenological scalar stiffness sector into a controlled micro-to-macro construction, sharp enough to be constrained, extended, or falsified.

## Figures and Tables

**Table 1:** SI dimensions and conventions used in the continuum EFT interface. The key point is that Φ(Θ) is an SI stiffness controlling the gravitational normalization; $F\left(\Theta \right)=\Phi /\Phi_{0}$ is dimensionless and defines the Weyl factor.

Symbol	Meaning	SI Dimension
*S*	Action	J·s
$x^{0}=ct,\;d^{4}x$	Relativistic coordinates/measure	$m,\;m^{4}$
$g_{\mu \nu },\;\sqrt{-g}$	Metric/volume factor	dimensionless
*R*	Ricci scalar	$m^{-2}$
*c*	Speed of light	$m\cdot s^{-1}$
*ℏ*	Quantum of action	J·s
$G_{N}$	Newton constant (reference)	$m^{3}\cdot {\mathrm{kg}}^{-1}\cdot s^{-2}$
Θ,χ,F,Ω,A	Phase/log/ratios/Weyl factors	dimensionless
$\Phi \left(\Theta \right),\;\Phi_{0}$	Jordan stiffness/reference stiffness	$\mathrm{kg}\cdot s^{-1}$
$K_{\Theta }\left(\Theta \right)$	Phase kinetic function in $-\frac{1}{2}K_{\Theta }{\left(\nabla \Theta \right)}^{2}$	$\mathrm{kg}\cdot s^{-1}$
V(Θ)	Potential term in the 4D action density	$J\cdot s\cdot m^{-4}$
cV(Θ)	Corresponding energy density (3D)	$J\cdot m^{-3}$
φ	Canonical scalar (Einstein frame)	${\left(\mathrm{kg}\cdot s^{-1}\right)}^{1/2}$
$\phi \equiv \varphi /\sqrt{\Phi_{0}}$	Dimensionless canonical field	dimensionless

## Data Availability

The original contributions presented in this study are included in the article. Further inquiries can be directed to the corresponding author.

## Abbreviations

The following abbreviations are used in this manuscript:

ADM	Arnowitt-Deser-Misner method
BEC	Bose-Einstein Condensate
EFT	Effective Field Theory
IR	Infrared (low-energy)
LogSE	Logarithmic Schrödinger Equation
RTQ	Relational Tension Quanta
SVT	Superfluid Vacuum Theory

## Appendix A. Controlled Induced-Gravity Derivation of $\frac{1}{2}$Φ(Θ)*R* from RTQ Microdynamics

### Appendix A.1. Goal and Strategy

We provide a controlled route from an explicit RTQ microdynamics to an induced Jordan frame term

(A1) $$S_{\mathrm{ind}}⊃\frac{1}{2}\int d^{4}x\sqrt{-g}\;\Phi \left(\Theta \right)\;R,$$

with a stiffness dependence Φ(Θ)∝cosΘ on the viable branch cosΘ>0. The derivation follows standard induced-gravity logic: integrate out a set of fast RTQ micro-fluctuations propagating on the ordered graph. In the graph-to-manifold limit, the resulting functional determinant generates local curvature terms in a derivative expansion. The key model input is that the fast-sector spectral cutoff (equivalently the proper-time UV regulator) is stiffness-controlled by the real channel $\Lambda^{P}=\Lambda_{0}\cos\Theta$.

### Appendix A.2. Explicit Microdynamics: Slow Phase *Θ*_i_ Plus Fast Internal Modes $\xi_{i}^{a}$

Consider an RTQ adjacency graph G=(V,E) with microscopic spacing $\ell_{0}$ (graph grain). Each node i∈V carries a compact phase $\Theta_{i}$ and complex tension $\Lambda_{i}=\Lambda_{0}e^{i\Theta_{i}}$ with real projection $\Lambda_{i}^{P}=\Lambda_{0}\cos\Theta_{i}$.

We now specify a concrete fast sector: $N_{\xi }$ real scalar internal modes $\xi_{i}^{a}$ ($a=1,\ldots ,N_{\xi }$), living on nodes and coupled to the ordered network. Their discrete action is chosen as a weighted graph Gaussian,

(A2) $$S_{\xi }\left[\xi ;\Theta \right]=\frac{1}{2}\sum_{a=1}^{N_{\xi }}\left[\sum_{\langle i,j\rangle \in E}\kappa_{ij}\left(\Theta \right)\;{\left(\xi_{i}^{a}-\xi_{j}^{a}\right)}^{2}+\sum_{i\in V}M^{2}\left(\Theta_{i}\right)\;{\left(\xi_{i}^{a}\right)}^{2}\right].$$

We adopt the minimal stiffness-controlled weights

(A3) $$\kappa_{ij}\left(\Theta \right)=\kappa_{0}\;\frac{\cos\Theta_{i}+\cos\Theta_{j}}{2\cos\Theta_{\mathrm{struct}}},\;M^{2}\left(\Theta_{i}\right)=M_{0}^{2}\;\frac{\cos\Theta_{i}}{\cos\Theta_{\mathrm{struct}}},$$

with $\kappa_{0}>0$ and $M_{0}^{2}>0$ fixed constants. On the EFT branch cosΘ>0, these choices ensure positivity and make the fast-sector stiffness explicitly proportional to cosΘ.

#### Interpretation

$\kappa_{ij}\left(\Theta \right)$ is a stiffness dependent conductance for fast-mode diffusion on links. $M^{2}\left(\Theta_{i}\right)$ is a local stiffness dependent micro-gap. Both are controlled only by the real channel cosΘ, consistent with the idea that the imaginary channel stores coherence but does not directly set metric rigidity.

### Appendix A.3. Graph-to-Manifold Limit and Emergence of a Laplace-Type Operator

Assume an ordered regime on scales $L\gg \ell_{0}$ in which:

(A4) $$\begin{array}{cc} |\Theta_{i}-\Theta_{j}| & \ll 1\;\mathrm{on}\;\mathrm{nearest}\;\mathrm{neighbors},\\ \Theta_{i} & ≃\Theta (x_{i})\;\mathrm{is}\;\mathrm{smooth}\;\mathrm{over}\;\mathrm{patches}\;\mathrm{of}\;\mathrm{size}\;L. \end{array}$$

Assume further that the weighted graph Laplacian associated with $\kappa_{ij}\left(\Theta \right)$ converges to a smooth Laplace-Beltrami operator on an emergent manifold $\left(M,g_{\mu \nu }\right)$ with an error controlled by $\ell_{0}/L$,

(A5) $$\Delta_{G}^{\left(\kappa \right)}\;⟶\;\nabla_{\mu }\;\left(\kappa \left(\Theta \right)\nabla^{\mu }\right)\;\mathrm{with}\;\mathrm{relative}\;\mathrm{error}\;\varepsilon_{\mathrm{cg}}∼O\;\left(\frac{\ell_{0}^{2}}{L^{2}}\right).$$

In that limit, the discrete action (A2) becomes

(A6) $$S_{\xi }≃\frac{1}{2}\sum_{a=1}^{N_{\xi }}\int d^{4}x\sqrt{-g}\;\left[\kappa \left(\Theta \right)\;g^{\mu \nu }\partial_{\mu }\xi^{a}\;\partial_{\nu }\xi^{a}+M^{2}\left(\Theta \right)\;{\left(\xi^{a}\right)}^{2}\right],$$

where κ(Θ) and $M^{2}\left(\Theta \right)$ are the coarse-grained versions of (A3):

(A7) $$\kappa \left(\Theta \right)=\kappa_{0}\;\frac{\cos\Theta }{\cos\Theta_{\mathrm{struct}}},\;M^{2}\left(\Theta \right)=M_{0}^{2}\;\frac{\cos\Theta }{\cos\Theta_{\mathrm{struct}}}.$$

#### Appendix A.3.1. Canonical Laplace-Type Form (Controlled Approximation)

To apply heat-kernel machinery, we rewrite (A6) in Laplace-type form. Define the field redefinition $\xi^{a}=\kappa {\left(\Theta \right)}^{-1/2}\;{\hat{\xi }}^{a}$. Up to terms containing derivatives of κ(Θ), one obtains

(A8) $$S_{\xi }≃\frac{1}{2}\sum_{a=1}^{N_{\xi }}\int d^{4}x\sqrt{-g}\;{\hat{\xi }}^{a}\left[-\nabla^{2}+m^{2}\left(\Theta \right)+\Delta_{\kappa }\left(\Theta ,\nabla \Theta \right)\right]{\hat{\xi }}^{a},$$

with the effective mass

(A9) $$m^{2}\left(\Theta \right)\equiv \frac{M^{2}\left(\Theta \right)}{\kappa (\Theta )}=\frac{M_{0}^{2}}{\kappa_{0}},$$

and where $\Delta_{\kappa }$ collects derivative terms generated by the rescaling (schematically combinations of ${\left(\nabla \ln\kappa \right)}^{2}$ and $\nabla^{2}\ln\kappa$). These terms contribute to the IR kinetic sector for Θ and are consistently absorbed into the EFT function $K_{\Theta }\left(\Theta \right)$ at the scalar-tensor level.

#### Appendix A.3.2. Where the Θ-Dependence Enters in This Controlled Template

With the minimal choice (A7), the ratio $m^{2}\left(\Theta \right)=M^{2}\left(\Theta \right)/\kappa \left(\Theta \right)$ is constant. In the present “controlled” construction, the leading Θ-dependence of the induced Einstein–Hilbert coefficient therefore enters through the stiffness-controlled UV scale (proper-time lower bound) in Appendix A.4, i.e., through $t_{\min}\left(\Theta \right)\propto \kappa {\left(\Theta \right)}^{-1}$ rather than through $m^{2}\left(\Theta \right)$. More general microscopic choices where $M^{2}\left(\Theta \right)$ and κ(Θ) scale differently would additionally induce Θ-dependence through the local endomorphism E(Θ) in the heat-kernel coefficients, but this is not needed to establish the robust Φ(Θ)∝cosΘ dependence in the present route.

#### Appendix A.3.3. Control Condition for Dropping Δ*_κ_* in the Gravity Sector

For the purpose of extracting the leading induced curvature term, it is sufficient that the stiffness varies slowly compared to the UV scale (see next subsection):

(A10) $$\frac{\left|\nabla \Theta \right|}{\Lambda_{\mathrm{UV}}}\ll 1,\;\frac{|\nabla^{2}\Theta |}{\Lambda_{\mathrm{UV}}^{2}}\ll 1,$$

so that $\Delta_{\kappa }$ contributes only to higher-order operators suppressed by $\Lambda_{\mathrm{UV}}^{-2}$.

### Appendix A.4. Functional Determinant and Proper-Time Regularization with Stiffness-Controlled UV Scale

The fast modes generate the effective action

(A11) $$e^{-\Gamma_{\xi }\left[g,\Theta \right]}=\int D\hat{\xi }\;\exp\;\left[-S_{\xi }\left[\hat{\xi };g,\Theta \right]\right]\propto {\left[\det D(\Theta ,g)\right]}^{-N_{\xi }/2},\;\Gamma_{\xi }=\frac{N_{\xi }}{2}\;\mathrm{Tr}\ln D,$$

with the Laplace-type operator from (A8):

(A12) $$D\left(\Theta ,g\right)≃-\nabla^{2}+m^{2}+\cdots .$$

Note that Equation (A12) is the operator of a time-independent Klein-Gordon equation, i.e., a relativistic Schrödinger equation for spin 0 particles (in flat Minkowski space). We can see how the dilaton field contribution looks like a relativistic bosonic field and therefore why the phenomenological BEC formulation of Modanese (Equations (8)–(10), [8]) as previously mentioned has some similarity to our dilatonic field action originally justified by dimensional scaling. We regulate TrlnD in proper time:

(A13) $$\Gamma_{\xi }=-\frac{N_{\xi }}{2}\int_{t_{\min}\left(\Theta \right)}^{\infty }\frac{dt}{t}\;\mathrm{Tr}\;e^{-tD}.$$

#### Stiffness-Controlled UV Cutoff (Explicit Model Input)

On a weighted graph, the diffusion time step corresponding to one microscopic hop scales as $t∼\ell_{0}^{2}/\kappa \left(\Theta \right)$ (higher conductance means faster diffusion). Therefore the minimal resolvable proper time in the continuum heat-kernel matching is

(A14) $$t_{\min}\left(\Theta \right)\equiv \frac{\ell_{0}^{2}}{\zeta \;\kappa (\Theta )},\;⟺\;\Lambda_{\mathrm{UV}}^{2}\left(\Theta \right)\equiv \frac{1}{t_{\min}\left(\Theta \right)}=\frac{\zeta \;\kappa (\Theta )}{\ell_{0}^{2}},$$

where ζ=O(1) encodes regulator conventions and graph details. With (A7), this implies

(A15) $$\Lambda_{\mathrm{UV}}^{2}\left(\Theta \right)=\Lambda_{\mathrm{UV}}^{2}\left(\Theta_{\mathrm{struct}}\right)\;\frac{\cos\Theta }{\cos\Theta_{\mathrm{struct}}}.$$

This single relation is what makes Φ(Θ)∝cosΘ emerge in a controlled way.

### Appendix A.5. Heat-Kernel Expansion and Extraction of the Induced *Φ(Θ)*R Term

In 4D, the heat-kernel asymptotics for a scalar Laplace-type operator reads

(A16) $$\mathrm{Tr}\;e^{-tD}∼\frac{1}{{\left(4\pi t\right)}^{2}}\int d^{4}x\sqrt{-g}\;\left[1+\frac{t}{6}R+O\left(t^{2}\right)\right],$$

up to standard convention choices and derivative-suppressed terms already discussed.

#### Appendix A.5.1. Vacuum-Energy Term and Scope

The leading constant term in (A16) also induces a large vacuum-energy contribution $\int d^{4}x\sqrt{-g}\;\Lambda_{\mathrm{vac}}$ (typically $\propto t_{\min}^{-2}$ in cutoff schemes). As usual in induced-gravity settings, its cancellation is a separate problem and is absorbed into the renormalized cosmological term of the EFT interface.

Keeping only the universal curvature piece and inserting (A16) into (A13), one finds

(A17) $$\begin{array}{cc} \Gamma_{\xi } & ⊃-\frac{N_{\xi }}{2}\int_{t_{\min}}^{\infty }\frac{dt}{t}\;\frac{1}{{\left(4\pi t\right)}^{2}}\int d^{4}x\sqrt{-g}\;\left(\frac{1}{6}R\right)t\\ \; & =-\frac{N_{\xi }}{12}\frac{1}{{\left(4\pi \right)}^{2}}\int d^{4}x\sqrt{-g}\;R\int_{t_{\min}}^{\infty }dt\;t^{-2}\\ \; & =-\frac{N_{\xi }}{12}\frac{1}{{\left(4\pi \right)}^{2}}\int d^{4}x\sqrt{-g}\;R\;\frac{1}{t_{\min}\left(\Theta \right)}. \end{array}$$

Using (A14), we identify the induced Jordan frame coefficient as

(A18) $$\begin{array}{cc} \Phi_{\mathrm{ind}}\left(\Theta \right) & =c_{\Phi }\;N_{\xi }\;\Lambda_{\mathrm{UV}}^{2}\left(\Theta \right)\\ \; & =c_{\Phi }\;N_{\xi }\;\Lambda_{\mathrm{UV}}^{2}\left(\Theta_{\mathrm{struct}}\right)\;\frac{\cos\Theta }{\cos\Theta_{\mathrm{struct}}},\\ c_{\Phi } & \equiv \frac{1}{6{\left(4\pi \right)}^{2}}\times O\left(1\right), \end{array}$$

where the overall O(1) factor absorbs regulator/sign conventions (Euclidean vs. Lorentzian matching) and the precise definition of ζ in (A14). The key point is the controlled functional dependence:

(A19) $$\Phi_{\mathrm{ind}}\left(\Theta \right)\;\propto \;\Lambda_{\mathrm{UV}}^{2}\left(\Theta \right)\;\propto \;\kappa \left(\Theta \right)\;\propto \;\cos\Theta \;\left(\cos\Theta >0\right).$$

#### Appendix A.5.2. Spectral Justification (Why $t_{\min}(\Theta )\propto \ell_{0}^{2}/\kappa (\Theta )$ Is Not Ad Hoc)

The Θ-dependence of the proper-time lower bound is fixed by the UV edge of the weighted diffusion operator on the microscopic graph. The fast-sector kinetic term defines a weighted graph Laplacian $L^{\left(\kappa \right)}\left(\Theta \right)$, and the dimensionful operator entering the heat kernel is $D\left(\Theta \right)=\left(1/\ell_{0}^{2}\right)L^{\left(\kappa \right)}\left(\Theta \right)+M^{2}\left(\Theta \right)$. For any bounded degree graph, one has general bounds implying $\lambda_{\max}\left(D\right)∼O\left(1\right)\times \kappa \left(\Theta \right)/\ell_{0}^{2}$ in the ordered quasi-uniform regime. A graph-intrinsic minimal resolvable proper time is then $t_{\min}\left(\Theta \right)\equiv c_{t}/\lambda_{\max}\left(D\left(\Theta \right)\right)\propto \ell_{0}^{2}/\kappa \left(\Theta \right)$, up to O(1) convention factors. Equivalently, imposing a constant cutoff on the dimensionless spectrum of the rescaled operator $\tilde{D}=\left(\ell_{0}^{2}/\kappa \left(\Theta \right)\right)D$ yields the same relation. Details are given in Appendix B.

#### Appendix A.5.3. Renormalized Matching and Calibration at Θ_struct_

The full Jordan frame coefficient is

(A20) $$\Phi \left(\Theta \right)=\Phi_{\mathrm{ct}}+\Phi_{\mathrm{ind}}\left(\Theta \right),$$

where $\Phi_{\mathrm{ct}}$ is the counterterm/bare piece. We impose the EFT calibration condition

(A21) $$\Phi \left(\Theta_{\mathrm{struct}}\right)=\Phi_{0},$$

which fixes $\Phi_{\mathrm{ct}}$ for any given microscopic parameter set $\left(N_{\xi },\kappa_{0},\ell_{0},\zeta \right)$. After this calibration, the leading predicted variation is

(A22) $$\frac{\Phi \left(\Theta \right)-\Phi \left(\Theta_{\mathrm{struct}}\right)}{\Phi (\Theta_{\mathrm{struct}})}=\frac{\Phi_{\mathrm{ind}}\left(\Theta \right)-\Phi_{\mathrm{ind}}\left(\Theta_{\mathrm{struct}}\right)}{\Phi_{0}}≃\frac{\cos\Theta }{\cos\Theta_{\mathrm{struct}}}-1,$$

up to derivative-suppressed corrections quantified below.

### Appendix A.6. Quantified Domain of Validity and Controlled Error Budget

The derivation above is a controlled derivative expansion around an ordered phase. The approximation errors can be organized into three small parameters.

#### Appendix A.6.1. (i) Coarse-Graining (Graph-to-Manifold) Error

The convergence (A5) requires

(A23) $$\varepsilon_{\mathrm{cg}}∼O\;\left(\frac{\ell_{0}^{2}}{L^{2}}\right)\ll 1,$$

with *L* the coarse-graining scale (patch size) on which Θ and link statistics are approximately smooth.

#### Appendix A.6.2. (ii) Curvature Expansion Control

Heat-kernel locality requires that typical curvature invariants are small compared to the UV scale:

(A24) $$\varepsilon_{R}\equiv \frac{\left|R\right|}{\Lambda_{\mathrm{UV}}^{2}\left(\Theta \right)}\ll 1,\;\varepsilon_{R^{2}}\equiv \frac{|R_{\mu \nu \rho \sigma }R^{\mu \nu \rho \sigma }|}{\Lambda_{\mathrm{UV}}^{4}\left(\Theta \right)}\ll 1.$$

In this regime, higher-derivative gravity terms (e.g., $R^{2}$) are suppressed by $\Lambda_{\mathrm{UV}}^{-2}$ and the leading Einstein–Hilbert truncation is justified.

#### Appendix A.6.3. (iii) Slow-Variation (Stiffness-Gradient) Control

Dropping $\Delta_{\kappa }$ in the extraction of Φ(Θ)R requires stiffness gradients to be small at the UV scale:

(A25) $$\varepsilon_{\Theta }\equiv \frac{{\left|\nabla \Theta \right|}^{2}}{\Lambda_{\mathrm{UV}}^{2}\left(\Theta \right)}\ll 1,\;\varepsilon_{\nabla^{2}\Theta }\equiv \frac{|\nabla^{2}\Theta |}{\Lambda_{\mathrm{UV}}^{2}\left(\Theta \right)}\ll 1.$$

When these hold, the κ-derivative terms contribute primarily to the scalar kinetic function $K_{\Theta }\left(\Theta \right)$ and to higher-order operators, not to the leading Φ(Θ)R coefficient.

#### Appendix A.6.4. Summary of Controlled Result

Under (A23)–(A25), the induced-gravity calculation yields, to leading order,

(A26) $$\Phi \left(\Theta \right)=\Phi_{0}\;\frac{\cos\Theta }{\cos\Theta_{\mathrm{struct}}}\;+\;O\;\left(\varepsilon_{\mathrm{cg}}+\varepsilon_{R}+\varepsilon_{\Theta }\right)\times \Phi_{0},\;\left(\cos\Theta >0\right),$$

after renormalized matching at $\Theta_{\mathrm{struct}}$.

### Appendix A.7. Remarks and Limitations

(1) The overall normalization of $\Phi_{\mathrm{ind}}$ is scheme dependent (proper-time cutoff vs. dimensional regularization). This is why the matching condition (A21) is essential. What is robust here is the functional dependence $\Phi \left(\Theta \right)\propto \Lambda_{\mathrm{UV}}^{2}\left(\Theta \right)\propto \kappa \left(\Theta \right)\propto \cos\Theta$ given the explicit micro-input (A14).
(2) The derivation does not assume a fundamental continuum metric. It assumes only that an ordered RTQ regime admits a graph-to-manifold limit for diffusion operators. Once that limit exists, the curvature expansion is standard and controlled by (A24).
(3) The breakdown near cosΘ→0 is explicit: $\Lambda_{\mathrm{UV}}^{2}\left(\Theta \right)\propto \cos\Theta$ collapses, and the conditions (A24)–(A25) cannot be maintained. This is the precise sense in which the continuum EFT fails before any divergence is physically meaningful.

## Appendix B. Spectral Origin of the Stiffness-Controlled UV Scale

### Appendix B.1. Weighted Graph Laplacian and Dimensionful Operator

Consider the fast-sector kinetic term on a weighted adjacency graph G=(V,E),

(A27) $$S_{\mathrm{kin}}\left[\xi \right]=\frac{1}{2}\sum_{\langle i,j\rangle \in E}\kappa_{ij}\left(\Theta \right)\;{\left(\xi_{i}-\xi_{j}\right)}^{2},$$

which defines the symmetric weighted graph Laplacian $L^{\left(\kappa \right)}\left(\Theta \right)$ through

(A28) $${\left(L^{\left(\kappa \right)}\xi \right)}_{i}=\sum_{j∼i}\kappa_{ij}\left(\Theta \right)\;\left(\xi_{i}-\xi_{j}\right).$$

In components,

(A29) $$L_{ij}^{\left(\kappa \right)}=\left\{\begin{array}{cc} -\kappa_{ij}\left(\Theta \right) & \mathrm{if}\;i\neq j\;\mathrm{and}\;i∼j,\\ \sum_{k∼i}\kappa_{ik}\left(\Theta \right) & \mathrm{if}\;i=j,\\ 0 & \mathrm{otherwise}. \end{array}\right.$$

The natural dimensionful operator entering the heat-kernel/proper-time representation is

(A30) $$D\left(\Theta \right)\equiv \frac{1}{\ell_{0}^{2}}\;L^{\left(\kappa \right)}\left(\Theta \right)+M^{2}\left(\Theta \right)\;1,$$

where $\ell_{0}$ is the microscopic spacing (graph grain). The UV spectral scale is then controlled by $\lambda_{\max}\left(D\right)$.

### Appendix B.2. Degree and Basic Bounds

Define the weighted degree at node *i*,

(A31) $$d_{i}^{\left(\kappa \right)}\left(\Theta \right)\equiv \sum_{j∼i}\kappa_{ij}\left(\Theta \right),\;d_{\max}^{\left(\kappa \right)}\left(\Theta \right)\equiv \max_{i\in V}d_{i}^{\left(\kappa \right)}\left(\Theta \right).$$

**Lemma** **A1** (UV edge of a weighted graph Laplacian)**.** *For any finite weighted graph Laplacian $L^{\left(\kappa \right)}\left(\Theta \right)$ defined above, one has*

(A32) $$d_{\max}^{\left(\kappa \right)}\left(\Theta \right)\;\leq \;\lambda_{\max}\;\left(L^{\left(\kappa \right)}\left(\Theta \right)\right)\;\leq \;2\;d_{\max}^{\left(\kappa \right)}\left(\Theta \right).$$

*In particular, the UV edge of the spectrum scales as $\lambda_{\max}\left(L^{\left(\kappa \right)}\right)∼O\left(1\right)\times d_{\max}^{\left(\kappa \right)}$.*

**Proof.** Upper bound. By Gershgorin’s theorem, every eigenvalue λ of $L^{\left(\kappa \right)}$ lies in at least one disk centered at the diagonal entry $L_{ii}^{\left(\kappa \right)}=d_{i}^{\left(\kappa \right)}$ with radius $\sum_{j\neq i}\left|L_{ij}^{\left(\kappa \right)}\right|=\sum_{j∼i}\kappa_{ij}=d_{i}^{\left(\kappa \right)}$. Hence $\lambda \leq d_{i}^{\left(\kappa \right)}+d_{i}^{\left(\kappa \right)}=2d_{i}^{\left(\kappa \right)}$ for some *i*, which implies $\lambda_{\max}\leq 2d_{\max}^{\left(\kappa \right)}$. Lower bound. Using the Rayleigh quotient for a real symmetric matrix,

(A33) $$\lambda_{\max}\;\left(L^{\left(\kappa \right)}\right)=\max_{x\neq 0}\frac{x^{T}L^{\left(\kappa \right)}x}{x^{T}x},\;x^{T}L^{\left(\kappa \right)}x=\sum_{\langle i,j\rangle \in E}\kappa_{ij}{\left(x_{i}-x_{j}\right)}^{2},$$

choose *x* to be the Kronecker vector localized at some node $i_{*}$: $x_{i_{*}}=1$ and $x_{i\neq i_{*}}=0$. Then $x^{T}x=1$ and

(A34) $$x^{T}L^{\left(\kappa \right)}x=\sum_{j∼i_{*}}\kappa_{i_{*}j}{\left(1-0\right)}^{2}=d_{i_{*}}^{\left(\kappa \right)}.$$

Taking $i_{*}$ that maximizes $d_{i}^{\left(\kappa \right)}$ yields $\lambda_{\max}\geq d_{\max}^{\left(\kappa \right)}$. Combining both bounds gives (A32). □

### Appendix B.3. Ordered Regime and Stiffness Scaling

In an ordered regime where Θ varies weakly on nearest neighbors, the weights are locally quasi-uniform. With the minimal choice used in the main derivation,

(A35) $$\kappa_{ij}\left(\Theta \right)=\kappa_{0}\frac{\cos\Theta_{i}+\cos\Theta_{j}}{2\cos\Theta_{\mathrm{struct}}},$$

one has on any coarse-graining patch P of size $L\gg \ell_{0}$,

(A36) $$\begin{array}{cc} \kappa_{ij}\left(\Theta \right)\; & =\kappa \left(\Theta_{P}\right)\;\left[1+O\left(\delta_{\Theta }\right)\right],\\ \kappa (\Theta_{P})\; & \equiv \kappa_{0}\frac{\cos\Theta_{P}}{\cos\Theta_{\mathrm{struct}}},\\ \delta_{\Theta }\; & ∼\max_{\langle i,j\rangle \subset P}\left|\Theta_{i}-\Theta_{j}\right|\ll 1. \end{array}$$

If the graph has bounded coordination number $\deg\left(i\right)\leq d_{\max}$ in the ordered phase, then

(A37) $$d_{i}^{\left(\kappa \right)}\left(\Theta \right)∼O\left(1\right)\times \deg\left(i\right)\;\kappa \left(\Theta_{P}\right)\;\Rightarrow \;d_{\max}^{\left(\kappa \right)}\left(\Theta \right)∼O\left(1\right)\times d_{\max}\;\kappa \left(\Theta_{P}\right),$$

up to $O(\delta_{\Theta })$ corrections. By Lemma A1,

(A38) $$\lambda_{\max}\;\left(L^{\left(\kappa \right)}\left(\Theta \right)\right)∼O\left(1\right)\times \kappa \left(\Theta_{P}\right).$$

Therefore, the dimensionful UV edge of D(Θ) satisfies

(A39) $$\lambda_{\max}\;\left(D(\Theta )\right)\;=\;\frac{1}{\ell_{0}^{2}}\lambda_{\max}\;\left(L^{\left(\kappa \right)}\left(\Theta \right)\right)+M^{2}\left(\Theta \right)\;∼\;O\left(1\right)\times \frac{\kappa (\Theta_{P})}{\ell_{0}^{2}},$$

where the additive $M^{2}\left(\Theta \right)$ does not affect the UV scaling.

### Appendix B.4. Exact Benchmark on a Regular Lattice (Optional but Useful)

On a *d*-dimensional hypercubic lattice with uniform nearest-neighbor weight $\kappa_{ij}=\kappa$, Fourier modes yield

(A40) $$\lambda \left(p\right)=2\kappa \sum_{\mu =1}^{d}\left(1-\cos(p_{\mu }\ell_{0})\right),$$

so the UV edge is reached at the Brillouin-zone corner $p_{\mu }\ell_{0}=\pi$ and is exactly

(A41) $$\lambda_{\max}\;\left(L^{\left(\kappa \right)}\right)=4d\;\kappa ,\;\lambda_{\max}\;\left(D\right)=\frac{4d}{\ell_{0}^{2}}\;\kappa +M^{2}.$$

This fixes the O(1) factor in (A39) in a canonical ordered-graph toy model.

### Appendix B.5. Proper-Time Lower Bound from the UV Spectral Edge

The discrete heat kernel admits the spectral representation

(A42) $$\mathrm{Tr}\;e^{-tD(\Theta )}=\sum_{n}e^{-t\lambda_{n}\left(D\left(\Theta \right)\right)}.$$

A conservative, graph-intrinsic notion of minimal resolvable proper time is obtained by requiring that the proper-time probe does not resolve structures beyond the UV spectral edge, i.e.,

(A43) $$t\;\geq \;t_{\min}\left(\Theta \right)\;\equiv \;\frac{c_{t}}{\lambda_{\max}\left(D\left(\Theta \right)\right)},\;c_{t}=O\left(1\right),$$

where $c_{t}$ encodes the precise cutoff profile (sharp/smooth). Using (A39) gives

(A44) $$t_{\min}\left(\Theta \right)\;∼\;O\left(1\right)\times \frac{\ell_{0}^{2}}{\kappa (\Theta_{P})},\;\Lambda_{\mathrm{UV}}^{2}\left(\Theta \right)\equiv \frac{1}{t_{\min}\left(\Theta \right)}∼O\left(1\right)\times \frac{\kappa (\Theta_{P})}{\ell_{0}^{2}}.$$

With κ(Θ)∝cosΘ on the viable branch cosΘ>0, this yields $\Lambda_{\mathrm{UV}}^{2}\left(\Theta \right)\propto \cos\Theta$.

### Appendix B.6. Equivalence to a Constant Cutoff on a Rescaled Operator

In the quasi-uniform regime (A36), define the dimensionless rescaled operator

(A45) $$\tilde{D}\left(\Theta \right)\;\equiv \;\frac{\ell_{0}^{2}}{\kappa (\Theta_{P})}\;D\left(\Theta \right)\;≃\;L^{\left(\hat{\kappa }\right)}+\frac{\ell_{0}^{2}M^{2}\left(\Theta \right)}{\kappa (\Theta_{P})}\;1,$$

whose UV spectral edge is O(1) (cf. (A41) when applicable). Then

(A46) $$\mathrm{Tr}\;e^{-tD(\Theta )}=\mathrm{Tr}\;e^{-\tau \;\tilde{D}\left(\Theta \right)},\;\tau \equiv t\;\frac{\kappa (\Theta_{P})}{\ell_{0}^{2}}.$$

Imposing a constant lower bound $\tau \geq \tau_{\min}$ on the proper-time parameter of the dimensionless operator $\tilde{D}$ is therefore equivalent to

(A47) $$t\;\geq \;t_{\min}\left(\Theta \right)=\tau_{\min}\;\frac{\ell_{0}^{2}}{\kappa (\Theta_{P})}.$$

Hence the Θ-dependence of $t_{\min}$ is not an independent regulator choice: it is the translation, in the unrescaled proper time *t*, of a constant spectral cutoff applied to the dimensionless UV edge of the underlying weighted diffusion operator.

## References

[1] Brans C., Dicke R.H. (1961). Mach’s Principle and a Relativistic Theory of Gravitation. Phys. Rev..

[2] Scott T.C., Zhang X., Mann R.B., Fee G.J. (2016). Canonical reduction for dilatonic gravity in 3 + 1 dimensions. Phys. Rev. D.

[3] Hirschman I.I. (1957). A Note on Entropy. Am. J. Math..

[4] Everett H. (1957). “Relative State” Formulation of Quantum Mechanics. Rev. Mod. Phys..

[5] Zloshchastiev K.G. (2025). Origins of logarithmic nonlinearity in the theory of fluids and beyond. Int. J. Mod. Phys. B.

[6] Scott T.C. (2023). From Modified Newtonian Dynamics to Superfluid Vacuum Theory. Entropy.

[7] Zloshchastiev K.G. (2020). An Alternative to Dark Matter and Dark Energy: Scale-Dependent Gravity in Superfluid Vacuum Theory. Universe.

[8] Modanese G. (1996). Theoretical analysis of a reported weak-gravitational-shielding effect. Europhys. Lett..

[9] Mann R.B., Ross S.F. (1993). The D to 2 limit of general relativity. Class. Quantum Gravity.

[10] Ohta T., Mann R.B. (1996). Canonical reduction of two-dimensional gravity for particle dynamics. Class. Quantum Gravity.

[11] Mann R.B., Ohta T. (1997). Exact solution for relativistic two-body motion in dilaton gravity. Class. Quantum Gravity.

[12] Renou M.O., Trillo D., Weilenmann M., Le T.P., Tavakoli A., Gisin N., Acín A., Navascués M. (2021). Quantum theory based on real numbers can be experimentally falsified. Nature.

[13] Zhai X.H., Zhao Y.B. (2005). A Cosmological model with complex scalar field. Nuovo C. Soc. Ital. Fis. A-Nucl. Part D.

[14] Yurov A.V. (2002). Complex field as inflaton and quintessence. arXiv.

[15] Migkas K., Pacaud F., Schellenberger G., Erler J., Nguyen-Dang N.T., Reiprich T.H., Ramos-Ceja M.E., Lovisari L. (2021). Cosmological implications of the anisotropy of ten galaxy cluster scaling relations. Astron. Astrophys..

[16] Secrest N.J., von Hausegger S., Rameez M., Mohayaee R., Sarkar S., Colin J. (2021). A test of the cosmological principle with quasars. Astrophys. J. Lett..

[17] Krishnan C., Mohayaee R., Colgáin E.O., Sheikh-Jabbari M., Yin L. (2022). Hints of FLRW breakdown from supernovae. Phys. Rev. D.

[18] Vaillant M. (2026). A single-flow model fails to account for localized low-z anisotropy in the Hubble residual field. Preprint.

[19] Li Z.D., Mao Y.L., Weilenmann M., Tavakoli A., Chen H., Feng L., Yang S.J., Renou M.O., Trillo D., Le T.P. (2022). Testing Real Quantum Theory in an Optical Quantum Network. Phys. Rev. Lett..

[20] Paliathanasis A. (2022). Complex scalar fields in scalar-tensor and scalar-torsion theories. Mod. Phys. Lett. A.

[21] Konopka T., Markopoulou F., Severini S. (2008). Quantum graphity: A model of emergent locality. Phys. Rev. D.

[22] Volovik G.E. (1999). Field theory in superfluid ^3^He: What are the lessons for particle physics, gravity, and high-temperature superconductivity?. Proc. Natl. Acad. Sci. USA.

[23] Volovik G.E. (2003). The Universe in a Helium Droplet.

[24] Castellanos E., Escamilla-Rivera C., Macías A., Núñez D. (2014). Scalar field as a Bose-Einstein condensate?. J. Cosmol. Astropart. Phys..

[25] Matos T., Castellanos E., Suárez A. (2017). Bose–Einstein condensation and symmetry breaking of a complex charged scalar field. Eur. Phys. J. C.

[26] Damour T., Esposito-Farèse G. (1992). Tensor-multi-scalar theories of gravitation. Class. Quantum Gravity.

[27] Uzan J.P. (2011). Varying Constants, Gravitation and Cosmology. Living Rev. Relativ..

[28] Damour T., Esposito-Farèse G. (1996). Tensor-scalar gravity and binary-pulsar experiments. Phys. Rev. D.

[29] Bertotti B., Iess L., Tortora P. (2003). A test of general relativity using radio links with the Cassini spacecraft. Nature.

[30] Williams J.G., Turyshev S.G., Boggs D.H. (2004). Progress in Lunar Laser Ranging Tests of Relativistic Gravity. Phys. Rev. Lett..

[31] Hofmann F., Müller J. (2018). Relativistic tests with lunar laser ranging. Class. Quantum Gravity.

[32] Hofmann F., Müller J., Biskupek L. (2010). Lunar laser ranging test of the Nordtvedt parameter and a possible variation in the gravitational constant. Astron. Astrophys..

[33] Adelberger E.G., Gundlach J.H., Heckel B.R., Hoedl S., Schlamminger S. (2009). Torsion balance experiments: A low-energy frontier of particle physics. Prog. Part. Nucl. Phys..

[34] Zloshchastiev K.G. (2011). Spontaneous symmetry breaking and mass generation as built-in phenomena in logarithmic nonlinear quantum theory. Acta Phys. Pol. B.

[35] Scott T.C., Zloshchastiev K.G. (2019). Resolving the puzzle of sound propagation in liquid helium at low temperatures. Low Temp. Phys..

[36] Scott T.C., Glasser M.L. (2025). Kink Soliton Solutions in the Logarithmic Schrödinger Equation. Mathematics.

[37] Sabín C., Bruschi D.E., Ahmadi M., Fuentes I. (2014). Phonon creation by gravitational waves. New J. Phys..

[38] Fernandez-Melendez H.A., Belyaev A., Gurzadyan V., Fuentes I. (2025). Probing lambda-gravity with Bose-Einstein condensates. Phys. Rev. Res..

